# High-fat diet increases the severity of *Giardia* infection in association with low-grade inflammation and gut microbiota dysbiosis

**DOI:** 10.1038/s41598-021-98262-8

**Published:** 2021-09-22

**Authors:** Thibault Allain, Elena Fekete, Olivia Sosnowski, Dimitri Desmonts de Lamache, Jean-Paul Motta, Dezirae Leger, Troy Feener, Raylene A. Reimer, André G. Buret

**Affiliations:** 1 Department of Biological Science, University of Calgary, Calgary, AB T2N 1N4 Canada; 2 Faculty of Kinesiology and the Department of Biochemistry and Molecular Biology, University of Calgary, Calgary, AB T2N 1N4 Canada; 3 IRSD, Université de Toulouse, INSERM, INRA, ENVT, UPS, U1220, CHU Purpan, CS60039, 31024 Toulouse, France

**Keywords:** Parasitology, Gastrointestinal diseases, Gastrointestinal diseases, Nutrition disorders, Microbiome

## Abstract

Exogenous factors that may influence the pathophysiology of *Giardia* infection remain incompletely understood. We have investigated the role of dietary fat in the pathogenesis of *Giardia* infection. Male 3 to 4-week-old C57BL/6 mice were fed either a low fat (LF) or a high fat (HF) diet for 12 days and challenged with *G. duodenalis*. In infected animals, the trophozoite burden was higher in HF + *Giardia* mice compared to the LF + *Giardia* group at day 7 post infection. Fatty acids exerted direct pro-growth effects on *Giardia* trophozoites. Analysis of disease parameters showed that HF + *Giardia* mice exhibited more mucosal infiltration by inflammatory cells, decreased villus/crypt ratios, goblet cell hyperplasia, mucus disruption, increased gut motility, and elevated fecal water content compared with LF + *Giardia*. HF diet-dependent exacerbation of *Giardia*-induced goblet cell hyperplasia was associated with elevated *Atoh1* and *Muc2* gene expression. Gut microbiota analysis revealed that the HF diet alone induces a taxonomic shift. HF + *Giardia* mice exhibited microbiota dysbiosis characterized by an increase of Firmicutes and a decrease of Bacteroidetes and significant changes in α- and β-diversity metrics. Taken together, the findings suggest that a HF diet exacerbates the outcome of *Giardia* infection. The data demonstrate that elevated dietary fat represents an important exogenous factor promoting the pathophysiology of giardiasis.

## Introduction

*Giardia duodenalis* (*syn G. lamblia* or *G. intestinalis*) is one of the most common enteric parasites and a leading cause of diarrheal disease worldwide, infecting 280 million people annually. It has been classified as a neglected disease by the World Health Organization (WHO)^1^. Most often, infection occurs via ingestion of food or water contaminated with *G. duodenalis* cysts, or by the fecal–oral route. Upon entering the proximal small intestine, *Giardia* cysts release the motile and proliferating trophozoites. When present, clinical manifestations of giardiasis are characterized by diarrhea, abdominal pain, nausea, intestinal malabsorption, and steatorrhea. *Giardia* infection may cause failure to thrive and impaired cognitive development in children. Moreover, individuals can develop post-infectious complications including irritable bowel syndrome (IBS) and chronic fatigue, as well as a variety of extra-intestinal abnormalities^2,3^.

The pathophysiology of giardiasis is multifactorial and remains incompletely understood^4–6^. *Giardia* cysteine proteases disrupt the intestinal tissue barrier, the mucus layers, and the gut microbiota, both at the site of infection and beyond^6–10^. These effects facilitate bacterial translocation. *Giardia* infection is responsible for maldigestion, and nutrient and water malabsorption^11^. Finally, increasing evidence suggests that the gut microbiota plays a key role in regulating both the colonization of trophozoites and the outcome of *Giardia* infection^10,12–18^. Indeed, *Giardia* fragments the microbiota biofilm and promotes the release of pathobionts that in turn trigger an inflammatory response^10^. Other reports indicate that *Giardia* trophozoites and/or excretory secretory products transform commensal bacteria into pathobionts that are lethal to *Caenorhabditis elegans*^19^.

Many factors influence the incidence and severity of parasitic diseases. Among these, diet plays a key role during the onset of parasitic infections^20^. Diet can directly modulate host resistance to parasites, affect parasitism through alterations to the microbiome, modulate parasite fitness, and directly or indirectly modulate host immunity^21^. Yet, the effects of diet on the pathophysiology of *Giardia* infection remain unclear. Previous reports observed that giardiasis may cause less severe disease in children living in low-income countries, where inflammatory diarrhea resulting from enteropathogenic bacterial infections is endemic^22–25^. Further research discovered that this was due at least in part to the degradation of pro-inflammatory cytokines by *Giardia* cathepsin B, which in turn attenuates the severity of inflammation induced by enteropathogenic bacteria^26,27^. In giardiasis, micronutrient deficiencies have been associated with increased disease severity in young individuals, while micronutrient and trace element supplementations have shown potential in reducing *Giardia*-associated diarrhea^23,28–33^. Other reports suggest that dietary fibers and prebiotic fibers, dietary proteins, and dietary fats may modulate *Giardia* infection^29,34–45^. In this context, we investigated the effects of a short-term Western diet, characterized by high fatty acids content, on the susceptibility to giardiasis. We hypothesized that Western diets may contribute to the persistence of *Giardia* in the gut in association with increased disease severity and microbiota dysbiosis.

## Results

### Animals fed a high fat diet are more susceptible to *G. duodenalis* and *G. muris* infections

**Figure 1:**
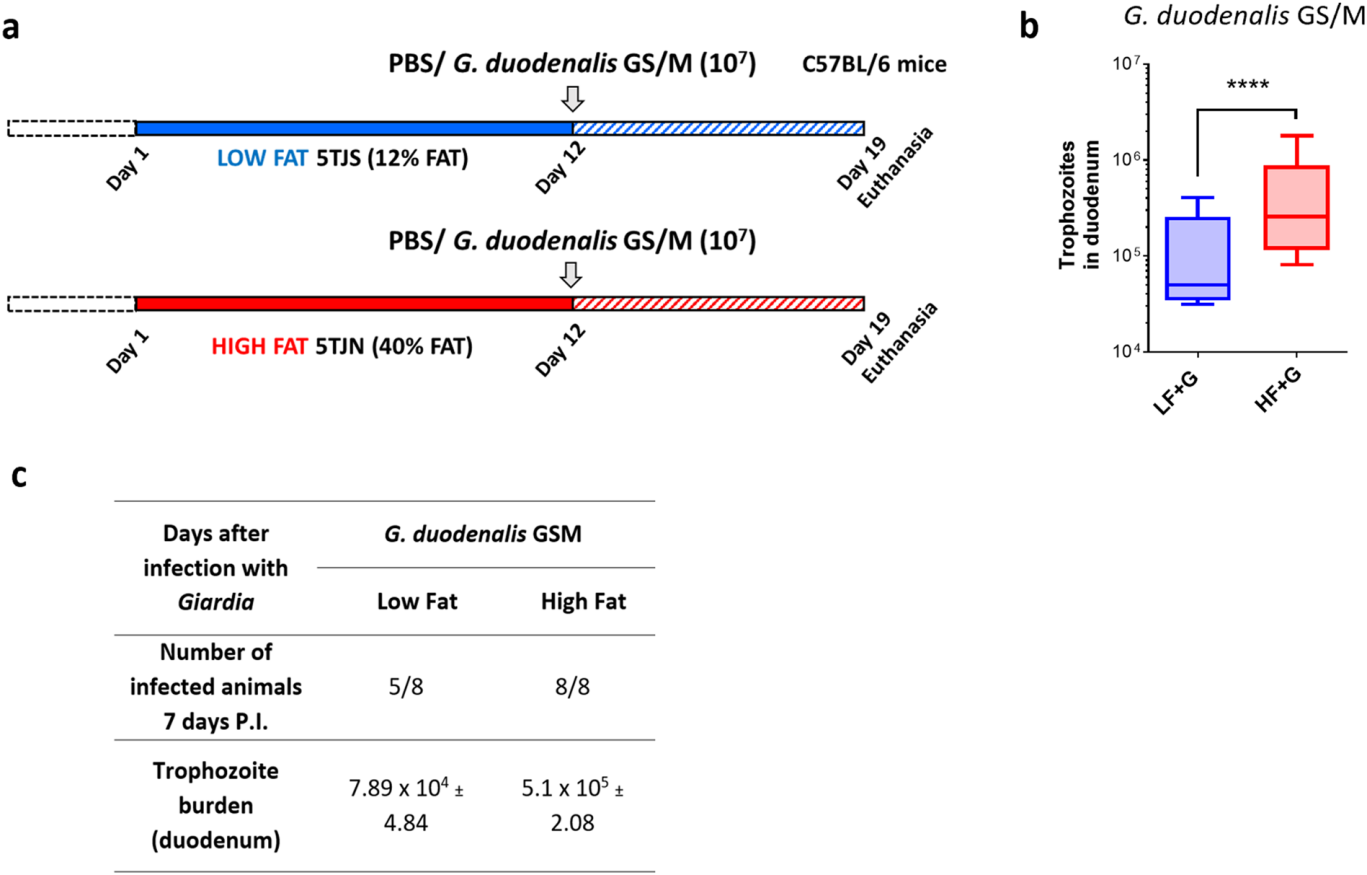
High fat diet increases host susceptibility to *Giardia* infection in C57BL/6 mice. (**a**) Experimental design (experimental condition #1). C57BL/6 mice (3–4-week-old) received either a low fat (LF) or a high fat (HF) diet for 12 days and were infected with *G. duodenalis* isolate GS/M (1 × 10^7^ trophozoites) at day 12. Control animals received PBS. Animals were euthanized at 7 days post-infection (day 19). (**b**) Duodenal *G. duodenalis* trophozoite burden was assessed at 7 days PI. Trophozoite burden was higher in HF + G mice compared with LF + G mice. No trophozoites were detected in the PBS group. (**c**) Number of infected animals per experimental group at 7 days PI. LF + G = low fat infected mice; HF + G = high fat infected mice. Data are representative of 8 mice/group and are shown as box plots with min/max whiskers; ****p < 0.001.

Male 3- to 4-week-old C57BL/6 mice were fed starting 12 days prior infection either a LF or HF diet. At day 12, animals were either infected with *G. duodenalis* GS/M (1 × 10^7^ trophozoites) or received PBS (Fig. 1a). At day 7 post-infection (PI), animals were sacrificed and trophozoite burden was determined in duodenal Sects. (3 cm of duodenum). In *Giardia*-infected groups, 3 out of 8 total LF + G mice had cleared the parasite at day 7 while 8 out of 8 total mice were still infected at day 7 in the HF + G group (Fig. 1a). The trophozoite burden was dramatically higher in HF + G mice (*p* < 0.001) compared with LF + G mice at day 7 PI (Fig. 1b). No cysts were detected in LF and HF + G duodenal sections at day 7 PI. No trophozoites were detected in the PBS group. Weight gain before infection (day 8) was significantly higher in HF mice compared to LF mice, however no significant differences were observed between LF and HF groups by day 12 (Fig. S1a, b). No significant differences in weight gain were observed in LF + G and HF + G groups compared to LF and HF groups, respectively (Fig. S1c). In a separate experiment (experimental setting #2), CD1 mice were fed LF and HF diets (identical formulation) for 3 weeks (Fig. S2a). At day 21, mice received either *Giardia muris* cysts (10^4^) or PBS. At day 7 PI, all mice were still infected (Fig. S2c).

The trophozoite burden was significantly higher in HF + G compared with LF + G mice (*p* < 0.05) (Fig. S2b). No cysts were detected in LF and HFG duodenal sections at day 7 PI. These results suggest that a short-term consumption of HF diet increases the host’s susceptibility to *Giardia* infection.

### HF diet promotes inflammatory cell infiltration and enhances mucosal damage in *Giardia*-infected mice

**Figure 2:**
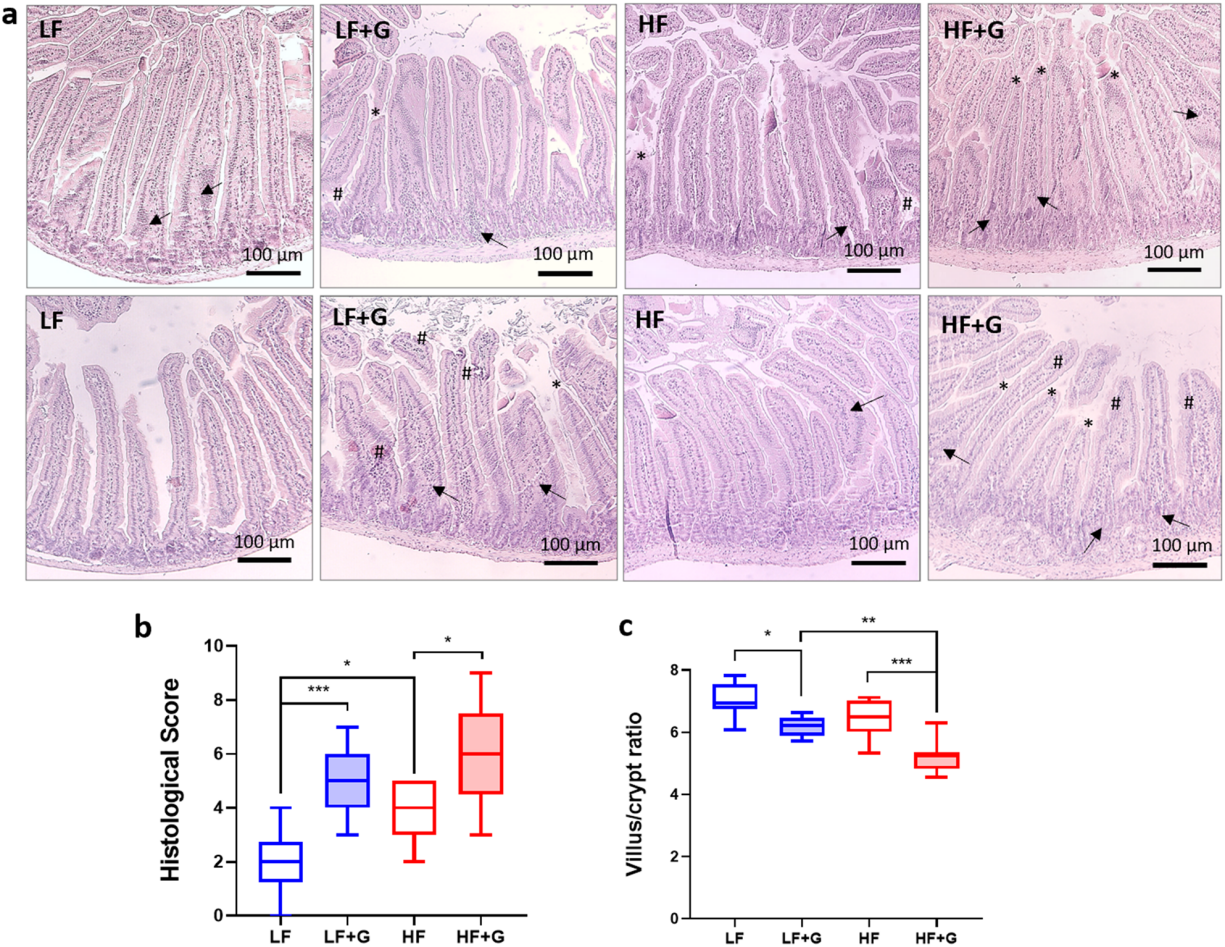
*Giardia* infection induces damage to the intestinal mucosa in a diet dependent manner. (**a**) H&E stained jejunal sections of control (LF and HF) and infected (LF + G and HF + G) C57BL/6 mice. Scale bar = 100 μm. (**b**) Histological score of the jejunum was determined on day 7 PI in control and infected animals (blind histological examination; scoring criteria: cell infiltration, cell hyperplasia, goblet cell loss, cryptitis, epithelial erosion, ulceration, tissue granulation, irregular crypts, crypt loss, villous damage, and blunting); n = 8/9 per group. (**c**) Morphometric measurements of the jejunal mucosa. Figure illustrates villus height to crypt depth ratios at day 7 post infection; n = 8–9 per group. Arrow (→) indicates cell infiltration; (#) indicates epithelial erosion; (*) indicates villous damage/blunting. LF = low fat control mice; LF + G = low fat infected mice; HF = high fat mice; HF + G = high fat infected mice. Data are shown as box plots with min/max whiskers; *p < 0.05, **p < 0.01, ***p < 0.001.

The HF diet, in the absence of infection, increased mucosal infiltration (both density and extent) of mixed inflammatory cells; types of inflammatory cell were not determined. Inflammatory cell infiltration scores in uninfected animals were significantly higher (*p* < 0.01) in the HF control group compared with the LF control group (Fig. 2a, b). Animals fed the HF diet also exhibited thicker jejunal *muscularis mucosae* compared with non-infected LF mice. Upon infection, LF + G mice showed a significant increase of jejunal inflammatory cell infiltration compared with LF control group (*p* < 0.001) (Fig. 2a, b). Mucosal and transmural inflammatory cell infiltration scores were also significantly increased in the HF infected group compared to HF controls (Fig. 2a, b). Cell infiltration was also observed in the submucosal layers of HF infected animals (Fig. 2a). Morphometric measurements of the proximal jejunal mucosa were performed by calculating villus height to crypt depth ratios at day 7 post infection (ImageJ software) (averaged from 5 to 10 crypts/tissue section). LF and HF uninfected mice showed similar villus/crypt ratios. Villus/crypt ratio was decreased in LF infected animals compared with LF controls (*p* < 0.05) (Fig. 2a, c). HF infected mice also showed a significant decrease of villus/crypt ratio compared with non-infected HF mice (*p* < 0.001). In addition, reductions of villus/crypt ratios were more severe in HF infected mice than in LF infected mice (Fig. 2a, c). Finally, both LF + G and HF + G animals showed signs of villus blunting (Fig. 2a). Together the results indicate that, in the *Giardia*-infected gut, a HF diet may promote the infiltration of inflammatory cells and exacerbate *Giardia*-induced mucosal damage.

### High fat diet-associated accumulation of pro-inflammatory cytokines is exacerbated upon *Giardia* infection

**Figure 3:**
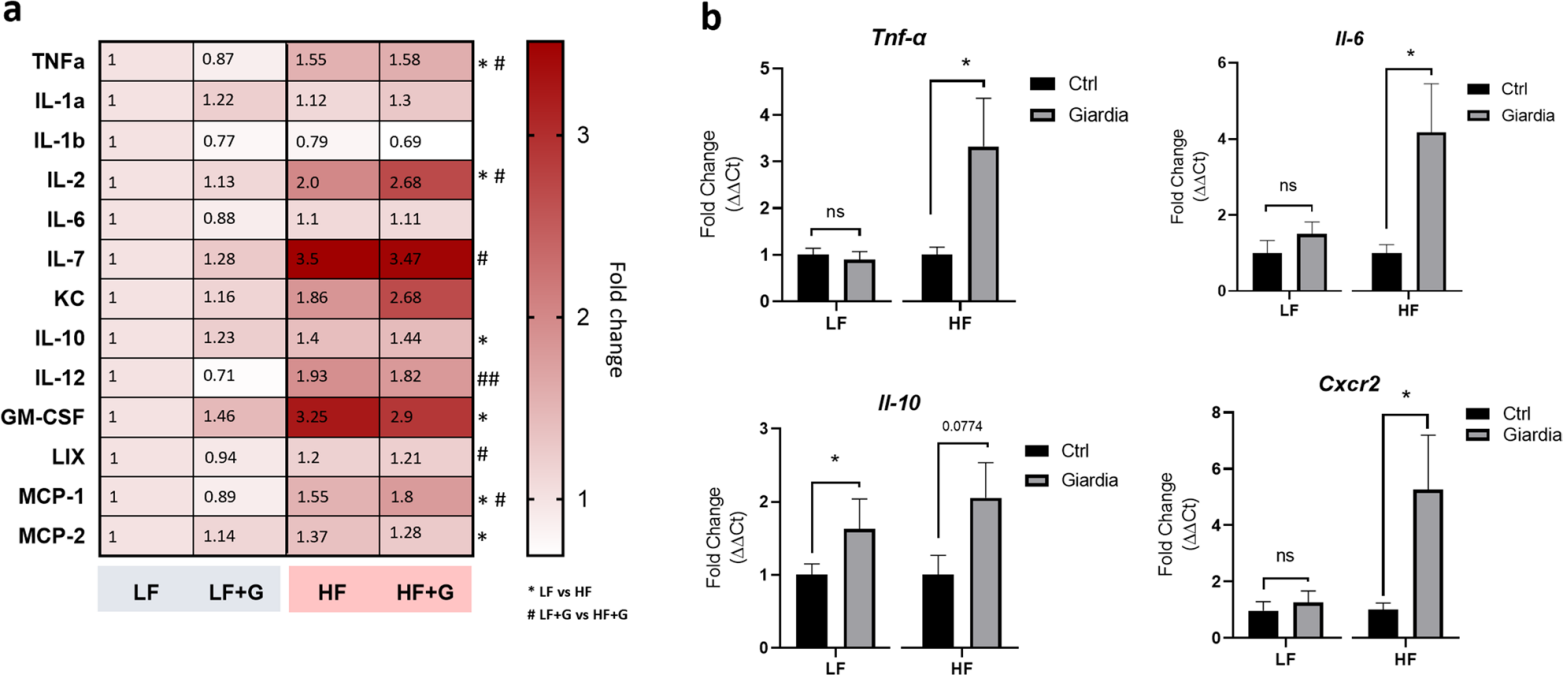
High fat diet exacerbates *Giardia*-induced low grade inflammation. (**a**) Multiplex analysis of jejunal tissue cytokines and chemokines assessed by Luminex Discovery Assay technology. The levels of 13 cytokines and chemokines were measured and represented as a heatmap. Data are represented as fold-change relative to uninfected low fat controls (range 0.5 to 3.25); n = 8–9/group. * *p* < 0.05 compared with LF control group; # *p* < 0.05 compared with LF + G group. (**b**) Jejunal mRNA levels of *Tnf-α, Il-6, Il-10* and *Cxcr2* were measured by Real-Time Quantitative Reverse Transcription-PCR and normalized to β-actin mRNA (2^-ΔΔCt^ method). Data are represented as fold change compared to LF and HF uninfected controls, respectively; n = 8–9/group. LF = low fat control mice; LF + G = low fat infected mice; HF = high fat mice; HF + G = high fat infected mice. Data are shown as mean ± S.E.M; *p < 0.05.

To further analyze the effects of the diets on mucosal inflammation upon *Giardia* infection, multiplex jejunal tissue cytokine and chemokine profiling was performed using Luminex Discovery Assay technology. The levels of 9 cytokines and 4 chemokines covering a large series of mechanisms involved in the mucosal immune response were measured and represented as a heatmap. In non-infected animals, HF diet was associated with an altered cytotoxic and chemotactic profile with significantly elevated levels of IL-2 (*p* < 0.05), TNF-α (*p* < 0.05), LIX (*syn.* CXCL5, RANTES) (*p* < 0.05), monocyte chemoattractant proteins MCP-1 (CCL2) (*p* < 0.05) and MCP-2 (CCL8) (*p* < 0.05), as well as granulocyte–macrophage colony-stimulating factor (GM-CSF) (*p* < 0.05), when compared to LF controls (Fig. 3a). Anti-inflammatory IL-10 levels were also elevated in these animals (*p* < 0.05). IL-7 (*p* = 0.053) and IL-12 (*p* = 0.075) were not significantly altered. IL-6, IL-1 -α and -β levels did not vary significantly between LF and HF uninfected mice (Fig. 3a). In mice given the LF diet, no significant differences in cytokines and chemokines profiles were observed between LF control and LF + G mice. The immune profile of the 3 LF + G mice that have fully cleared the infection did not very significantly from the 5 mice that were still infected at Day 7 PI. In contrast, a small but non-significant increase of pro-inflammatory cytokines such as IL-2, TNF-α and IL-12 was observed in HF + G groups versus uninfected HF mice (Fig. 3a). Elevated chemokine levels (GM-CSF, LIX, MCP-1) were similar in HF and HF + G mice (Fig. 3a). The increase of NF-κB regulated pro-inflammatory cytokines such as TNF-α and IL-6 was confirmed at the mRNA level by RT-qPCR. In LF animals, no increases in *Tnf-α* and *Il-6* were observed upon *Giardia* infection compared with non-infected controls (Fig. 3b). In HF conditions, *Giardia* infection resulted in increased expression of *Tnf-α* (3.3-fold; *p* < 0.05) and *Il-6* (4.2-fold; *p* < 0.05) compared with HF controls (*Il-2* and *Il-12* mRNA could not be detected in jejunal tissues) (Fig. 3b). Elevated mRNA expression of *Stat1* (*p* = 0.08) and *Gata-3* (*p* < 0.05), which are key transcription factors of Th1 and Th2 immunity respectively, was only observed in HF mice that were infected by *G. duodenalis* (Fig. S5a, b). Interestingly, Th2 and Th17 cytokines (*Il-4*, *Il-5*, *Il-13, Il-17A* and *Il-25*) mRNA were below detectable levels in all experimental groups (data not shown). Taken together, these observations suggest that a HF diet induces a low-grade NF-κB driven inflammatory response in the upper small intestine, which is exacerbated upon infection by *Giardia*. Additionally, elevated *Cxcr2* gene expression, a key regulator of neutrophil recruitment, was observed in HF + G mice but not in the other groups (5.2-fold*; p* < 0.05) (Fig. 3b). These data corroborate the mucosal inflammatory cell infiltration observed in histology sections.

### Fatty acids increase trophozoite metabolic activity and exacerbate disruption of tight junction proteins induced by *G. duodenalis*

**Figure 4:**
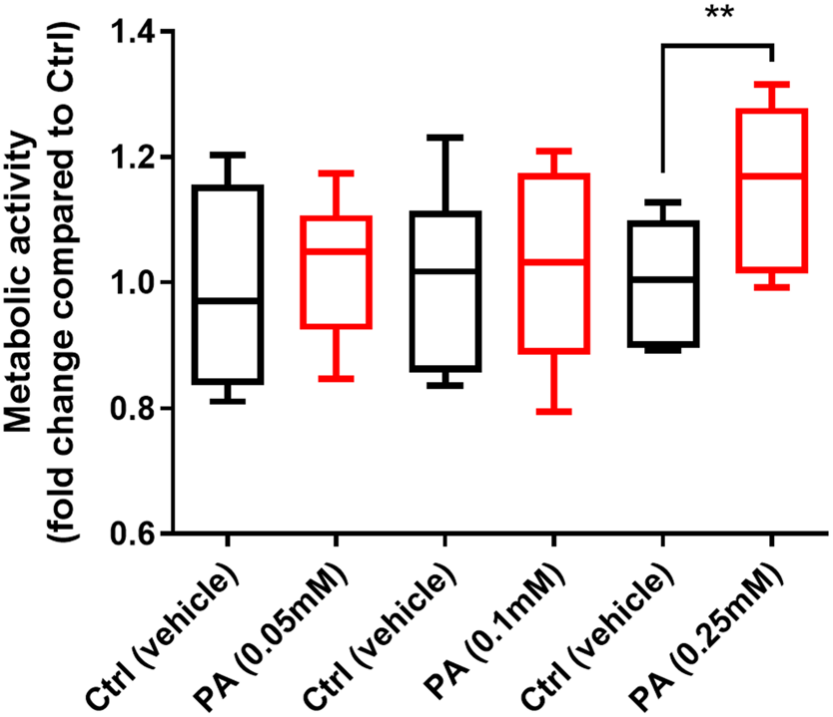
Palmitic acid enhances *Giardia* trophozoites’ metabolic activity. Levels of resorufin converted from resazurin by *G. duodenalis* trophozoites (isolate NF) at 37 °C for 90 min. Resorufin fluorescence was read at λexc = 550 nm; λem = 590 nm. Data are represented as fold-change compared to control. Ctrl = control (vehicle); PA = Palmitic acid. Data are shown as box plots with min/max whiskers; **p < 0.01.

To explore the direct role of HF diet components in promoting the growth of *Giardia* trophozoites, we then assessed the role of fatty acids in *Giardia* metabolic activity by measuring the red-ox balance via the conversion of resazurin into its reduced form resorufin. *G. duodenalis* NF trophozoites were incubated at 37 °C for 24 h with increasing concentrations of palmitic acid (PA) (0.05, 0.1 and 0.25 mM) and control trophozoites received vehicle (BSA). After 24 h, the trophozoite metabolic activity was enhanced in the presence of PA (0.25 mM) compared to vehicle (Fig. 4). Similar results were obtained in the presence of OA (0.25 mM) (Fig. S8). Altogether these results also indicate that dietary fatty acids such as PA (saturated) and OA (monounsaturated) may exert direct pro-growth effects on *Giardia* trophozoites.

**Figure 5:**
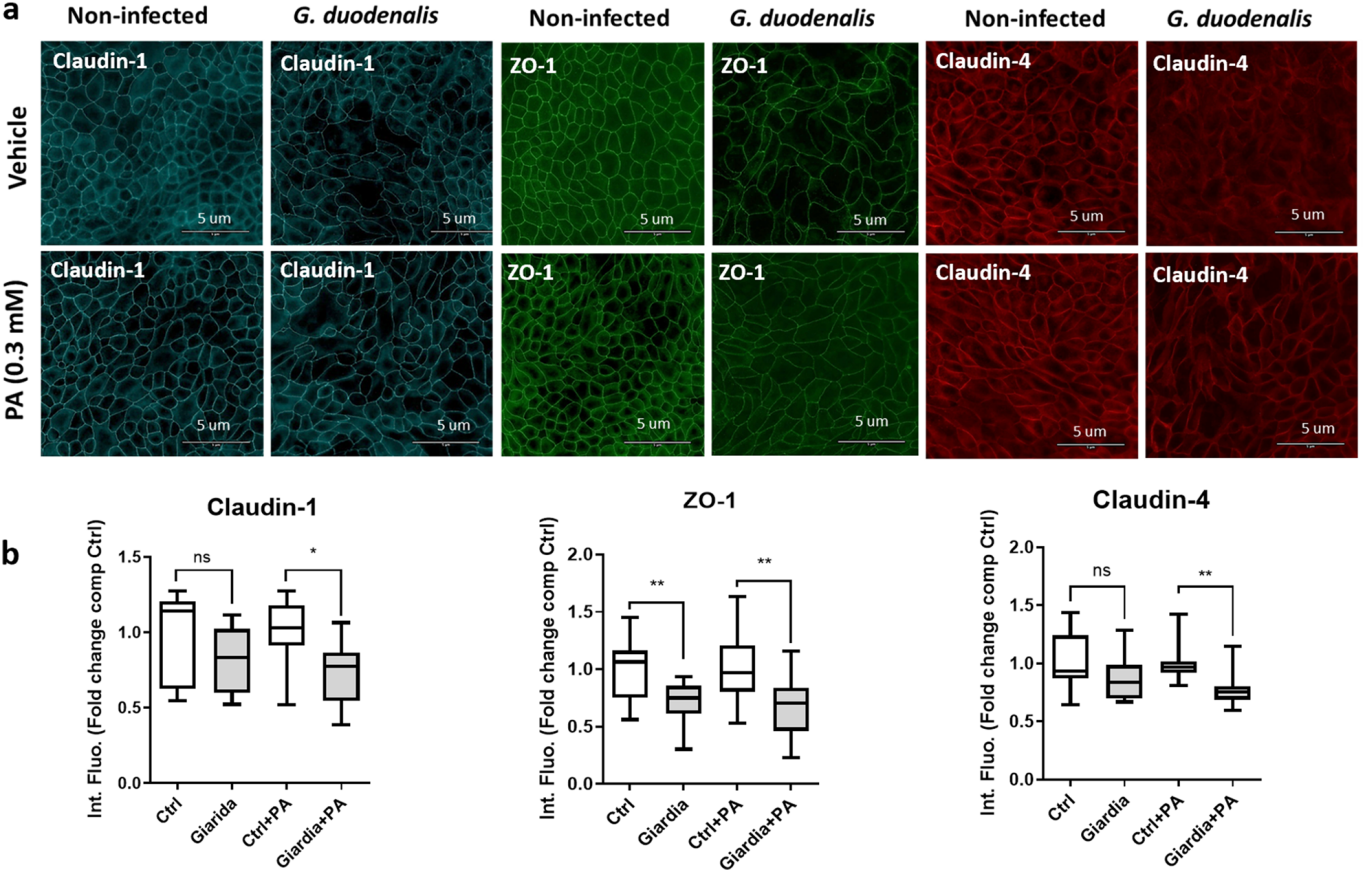
Disruption of tight junction proteins claudin-1 and claudin-4 by *Giardia* is enhanced in the presence of palmitic acid in SCBN cells. Intestinal epithelial cells (SCBN) were pre-treated with vehicle (Ctrl) or palmitic acid (PA; 0.3 mM) and challenged for 24 h with *G. duodenalis* isolate GS/M (*Giardia* + PA; MOI = 10:1) or left un-infected (Ctrl + PA). (**a**) SCBN cells were probed for immunofluorescence staining of ZO-1, claudin-1 and claudin-4 proteins (scale bar = 5 μm). (**b**) ZO-1, claudin-1 and claudin-4 relative protein levels were quantified by fluorescence intensity compared with DAPI (ImageJ) and expressed as fold change compared to Ctrl group. n = 4 per group. Data are expressed as box plots with min/max whiskers.*p < 0.05, **p < 0.01.

Increased epithelial permeability resulting from loss of barrier function is a central element of *Giardia* pathophysiology. In this context, we assessed the influence of diet components in the disruptions of tight junctional proteins including zonula occludens-1 (ZO-1), claudin-1 and claudin-4 by *G. duodenalis* GS/M. To determine the role of the dietary fats in *Giardia*-induced disruption of tight junction proteins, non transformed intestinal epithelial cells SCBN^46^ were cultivated in the presence or absence of palmitic acid, a widely used alimentary saturated fatty acid, for 2 h. Cells were then infected with *G. duodenalis* GS/M trophozoites for 24 h. Following incubation, ZO-1, claudin-1 and claudin-4 expressions were assessed via immunohistochemistry, and intensity of fluorescence was measured using ImageJ software. Palmitic acid alone (0.3 mM) did not modify the expression of claudin-1, claudin-4 and ZO-1 (Fig. 5a, b). A significant decrease of ZO-1 but not claudin-1 and claudin-4 expression was observed in *Giardia* infected cells without PA (Fig. 5a, b) (n = 4). In contrast, SCBN incubation with PA (0.3 mM) prior to infection resulted in a significant reduction of ZO-1, claudin-1 and claudin-4 expression compared to control cells exposed to PA (0.3 mM) alone (Fig. 5a, b) (n = 4). Noticeably, disruption and/or rearrangement of ZO-1, claudin-1 and claudin-4 was further exacerbated when cells were incubated with PA compared to untreated infected *Giardia*-infected SCBN cells, when compared to uninfected controls (Fig. 5a, b). Similar results were obtained when cells were incubated with OA (Fig. S7A, B). No significant differences were observed by directly comparing *Giardia* and *Giardia* + PA/or *Giardia* + OA groups (data not shown).

### High fat diet exacerbates *Giardia*-induced jejunal goblet cell hyperplasia and elevated *Muc2* and *Atoh1* gene expression

**Figure 6:**
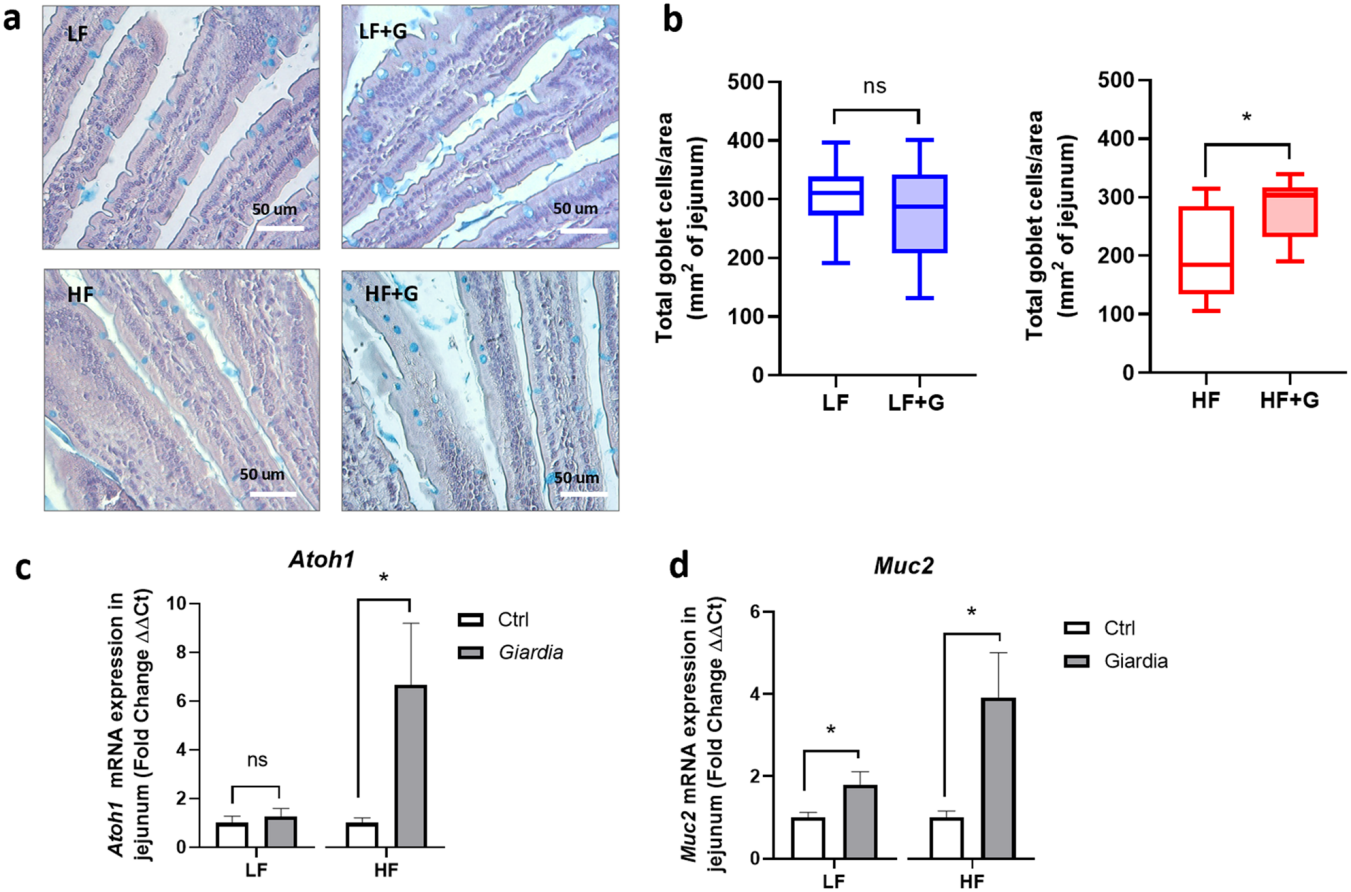
High fat exacerbates *Giardia*-induced alterations in goblet cell numbers and jejunal mucin gene expression. (**a**) Alcian Blue/PAS staining of jejunal sections of control (LF and HF) and infected (LF + G and HF + G) C57BL/6 mice. Goblet cells are stained in light blue. Scale bar = 50 μm. (**b**) Total goblet cell count per mm^2^ of jejunum. Surface area was quantified using ImageJ software. n = 8–9 per group. Data are expressed as box plots with min/max whiskers; *p < 0.05. (**c**) *Atoh1* mRNA transcript expression in the jejunum, normalized to β-actin mRNA (2^−ΔΔCt^ method); n = 8–9. Data are shown as mean ± S.E.M; *p < 0.05. (**d**) *Muc2* mRNA transcript expression in the jejunum, normalized to β-actin mRNA (2^-ΔΔCt^ method). n = 8–9. LF = low fat control mice; LF + G = low fat infected mice; HF = high fat mice; HF + G = high fat infected mice. Data are shown as mean ± S.E.M; *p < 0.05.

As mucus disruptions appear to play an important role in the pathophysiology of giardiasis^9^, diet-dependent effects of *G. duodenalis* on intestinal goblet cells were visualized in vivo in Carnoy’s fixed tissues. Alcian Blue/PAS staining of proximal jejunum tissue sections revealed that total goblet cells count per mm^2^ was significantly decreased in HF mice compared with LF mice (*p* < 0.05) (Fig. 6a, b). LF groups did not show differences between control and infected animals in total goblet cell counts (plain and depleted) (Fig. 6a, b). HF + G animals had a higher total goblet cell count per mm^2^ compared with HF (Fig. 6a, b). Consistent with these observations, the expression of *Atoh1*, a marker of intestinal secretory cell differentiation, was significantly increased in the HF + G group compared with the HF controls (Fig. 6c). *Atoh1* gene expression remained low in both LF control and infected mice, with no difference observed (Fig. 6c). These results suggest that *Giardia* induces goblet cell hyperplasia in a diet dependent manner. In the jejunum, the gene expression of *Muc2*, a mucin gene coding for the major intestinal gel-forming mucin MUC2, was significantly increased in HF + G animals compared to HF controls (*p* < 0.05) (Fig. 6d). This increase in *Muc2* expression was also observed in the LF infected group to a lesser extent (*p* < 0.05). Together, the results suggest that HF diet exacerbates *Giardia*-associated goblet cell hyperplasia in association with elevated *Atoh1* gene expression in the small intestine.

### *Giardia* infection is associated with mucus granule depletion in the colon

**Figure 7:**
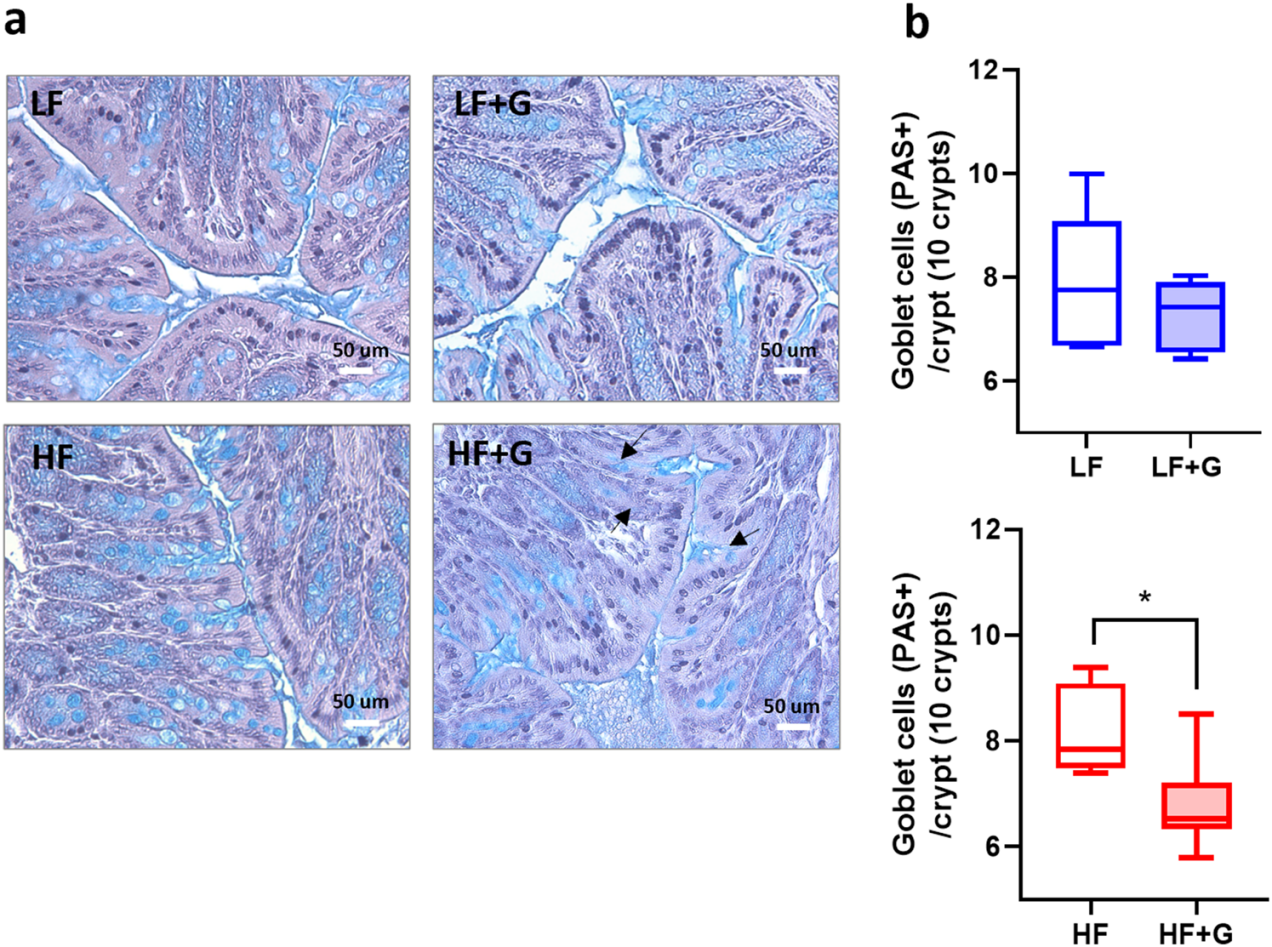
High fat exacerbates *Giardia*-induced depletion of colonic goblet cells. (**a**) Alcian Blue/PAS staining of distal colon sections of control (LF and HF) and infected (LF + G and HF + G) C57BL/6 mice. Goblet cells are stained in light blue. Scale bar = 50 μm. (**b**) Total goblet cell count (PAS +) per crypt. Blind histological examination 10 crypts of jejunum. Surface area was quantified using ImageJ software. Arrows indicate colonic goblet cells depletion. n = 8–9 per group. Data are expressed as box plots with min/max whiskers; *p < 0.05.

To explore the influence of diet on the effects of *G. duodenalis* infection in the colon, goblet cells and mucus granules were visualized in the colon. In Carnoy’s fixed embedded tissues PAS/AB staining showed no differences in mucus granule/goblet cells per colon crypt between LF and HF control mice (Fig. 7a, b). In LF mice, *Giardia* infection did not cause a depletion of intracellular mucin compared with non-infected animals. Notably, goblet cell counts per colon crypt were significantly decreased in *Giardia* HF animals in comparison to non-infected HF controls (*p* < 0.05) (Fig. 7a, b). The results suggest that *G. duodenalis*-associated stimulation of mucin release is enhanced in HF diet, resulting in goblet cell depletion. These results are consistent with recent observations suggesting that *Giardia* disrupts the mucus layers beyond the site of trophozoite colonization, *e.g.* in the colon^9^.

### Microbiota dysbiosis is enhanced in high fat diet mice infected with *Giardia*

The β-diversity metric showed distinct separation between LF and HF non-infected mice (F-value: 11.671; R-squared: 0.45465; p-value < 0.001) (data not shown). The α-diversity metrics such as Shannon and Simpson’s diversity metrics were not significantly different between LF and HF control mice (*p*-value = 0.115) (Fig. S9a, b). HF mice showed a significant increase of bacteria belonging to the Bacteroides and Verrucomicrobia phyla and a decrease of bacteria belonging to the Firmicutes phylum (Fig. S9d). Linear Discriminant Analysis (LDA) score calculated for differences at the Family level showed that the HF diet was associated with an increase of *Bacteroidaceae*, *Verrucomicrobiaceae*, *Peptostreptococcaceae*, *Rikenellaceae* and *Clostridiaceae* (LDA score > 3, *p* < 0.05), while the LF diet was positively correlated with an increase of *Alcaligenaceae*, *Ruminococcaceae*, *Lachnospiraceae* and *Porphyromonadaceae* (LDA score > 3, *p* < 0.05) (Fig.s S4a, S9c, d). Together, these data suggest that short-term consumption of dietary fat can directly induce significant shifts in the bacterial communities of the gut.

**Figure 8:**
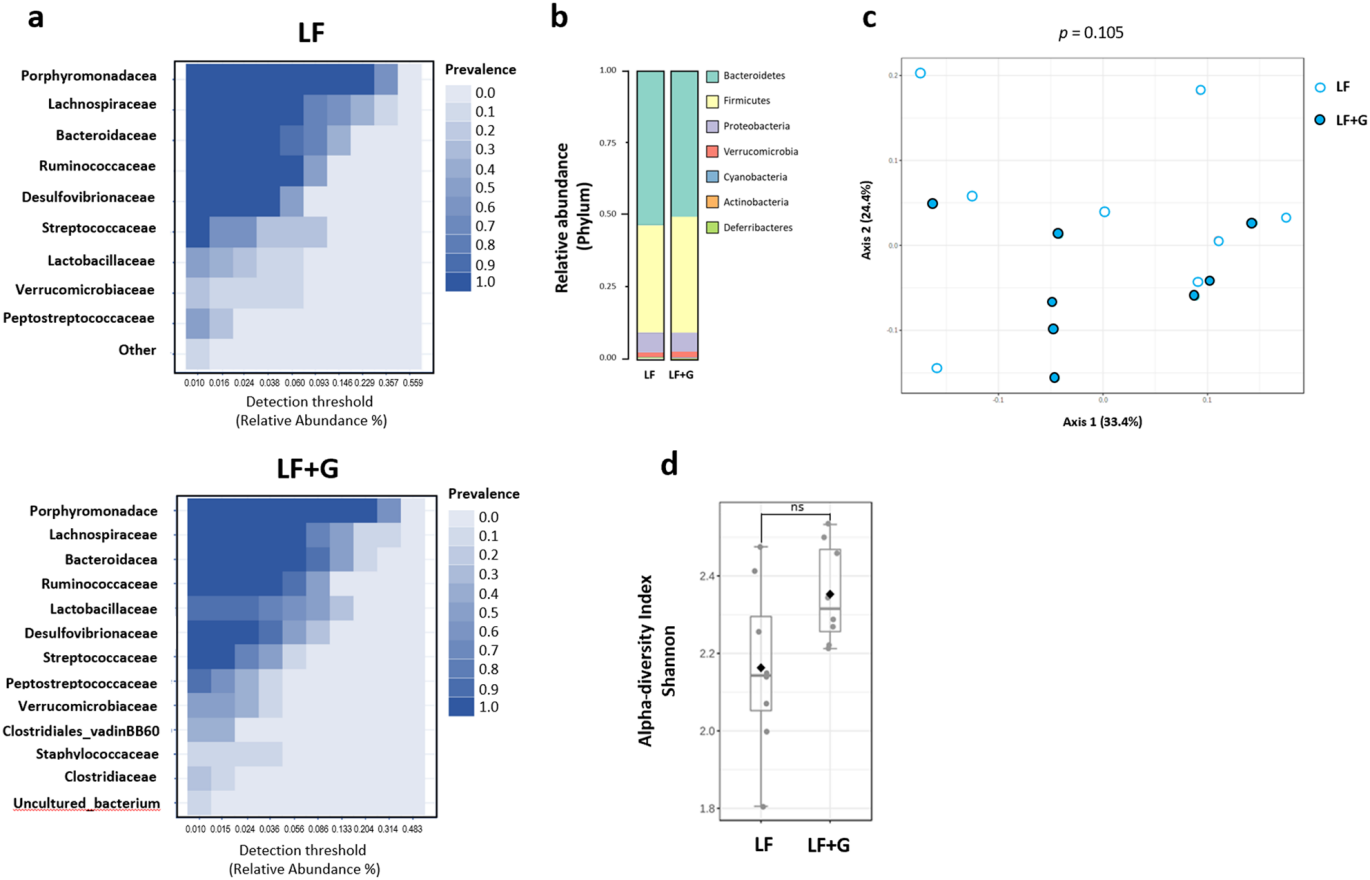
Microbiota composition is not significantly altered upon *Giardia* infection in mice fed a low-fat diet. The fecal microbiota composition of *Giardia*-infected LF + G group was investigated at day 7 PI and compared with LF control mice microbiota, respectively. Taxonomic identification of the gut microbiota was assessed via 16S rRNA gene sequencing using Illumina MiSeq platform. The 16S rRNA amplicons were clustered into operational taxonomic units (OTU) with a 97% identity threshold. Data are represented using Total Sum Scaling (proportional abundance of species) to remove sequencing-related technical biases. (**a**) Bacterial taxa at the family level based on their prevalence at a given abundance threshold (core microbiota representation; relative abundance %); prevalence scale ranges from 0.0 (white) to 1.0 (dark blue). (**b**) Bar charts representative of the relative abundance of microbial taxa at the phylum level between LF and LF + G groups. (**c**) The β-diversity between LF (white dots) and LF + G (blue dots) microbial communities was assessed using Bray–Curtis dissimilarity index and visualized through Principal Coordinate Analysis (PCoA) plot. (**d**) The α-diversity was assessed by calculating Shannon diversity index; data are expressed as box plots with min/max whiskers. n = 8 mice per group; LF = low fat control mice; LF + G = low fat infected mice.

Upon infection with *G. duodenalis*, mice given the LF diet did not exhibit any significant alteration of fecal microbiota composition at the phylum level compared with LF control mice (Fig. 8a). β-diversity indices did not show significant separation caused by the infection with *Giardia* in LF mice (F-value: 1.6828; R-squared: 0.1073; *p* < 0.105) (Fig. 8c). Similarly, α-diversity index (Shannon index) failed to show a difference of species richness at the genus level between the two groups (Fig. 8d). At the family level, both LF controls and LF infected mice showed similar abundances of (in order of importance) *Porphyromonadaceae* (Bacteroidetes), *Lachnospiraceae* (Firmicutes), *Bacteroidaceae* (Bacteroidetes) and *Ruminococcaceae* (Firmicutes) (Fig. 8a). Together, these data indicate that *Giardia* infection in animals fed a low-fat diet only causes modest taxonomic changes after 7 days of infection. Interestingly, a significant increase (LDA score > 5; *p* < 0.05) of lactobacilli (*Lactobacillaceae* family) was detected in LF + G mice only (Fig. S4b, d), a trend also observed in HF + G mice to a lesser extent (Fig. S4c).

**Figure 9:**
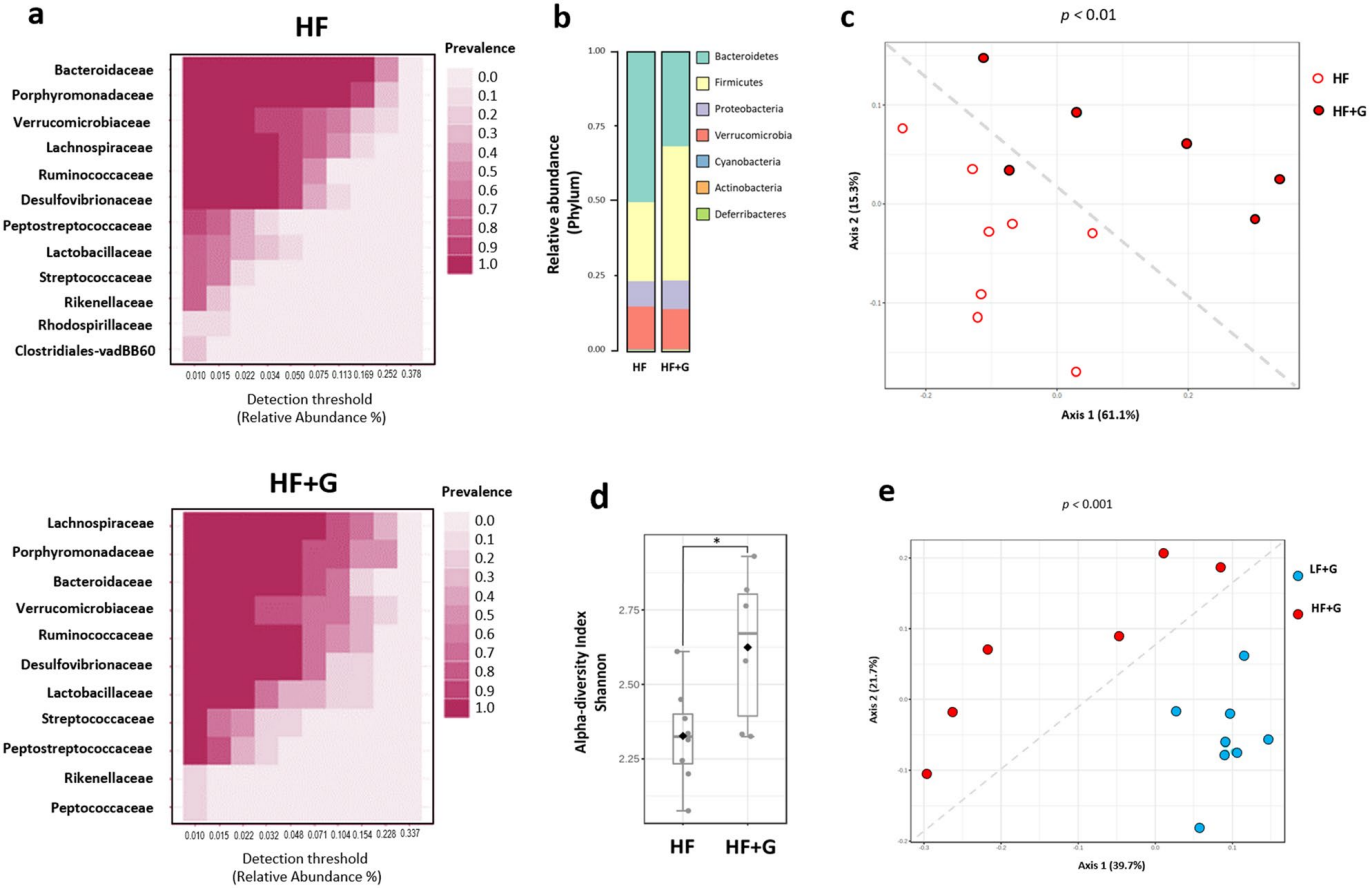
High fat significantly alters microbiota composition upon *Giardia* infection. The fecal microbiota composition of *Giardia*-infected HF + G group was investigated at day 7 PI and compared with HF control mice microbiota, respectively (6 to 8 mice per group). Taxonomic identification of the gut microbiota was assessed via 16S rRNA gene sequencing using Illumina MiSeq platform. The 16S rRNA amplicons were clustered into operational taxonomic units (OTU) with a 97% identity threshold. Data are represented using Total Sum Scaling (proportional abundance of species) to remove sequencing-related technical biases. (**a**) Bacterial taxa at the family level based on their prevalence at a given abundance threshold (core microbiota representation; relative abundance %); prevalence scale ranges from 0.0 (white) to 1.0 (dark fuchsia). (**b**) Bar charts representative of the relative abundance of microbial taxa at the phylum level between HF and HF + G groups. (**c**) The β-diversity between HF (white dots) and HF + G (red dots) microbial communities was assessed using Bray–Curtis dissimilarity index and visualized through Principal Coordinate Analysis (PCoA) plot. (**d**) The α-diversity was assessed by calculating Shannon diversity index. Data are expressed as box plots with min/max whiskers; *p < 0.05. (**e**) The β-diversity between LF + G (blue dots) and HF + G (red dots) microbial communities was assessed using Bray–Curtis dissimilarity index and visualized through PCoA plot. n = 6–8 mice per group; one sample from HF *Giardia* group was removed due to low abundance OTUs and failure to pass the OTU selection step. HF = high fat control mice; HF + G = high fat infected mice.

The analysis of the fecal microbiota of HF mice showed a dramatic taxonomic shift of bacterial communities driven by *Giardia* infection. Bray–Curtis dissimilarity index (β-diversity metric) showed a clear separation (F-value: 4.6235; R-squared: 0.27813; *p* < 0.01) between HF control and infected groups (Fig. 9c). Shannon and Simpson’s α-diversity indexes revealed a significant increase of the bacterial richness and evenness in HF infected mice as compared to HF controls (*p* < 0.05) (Fig. 9d). Relative abundance of bacterial species at the phylum level indicates a significant increase of Firmicutes (36% vs 26%) as well as a slight decrease of Bacteroidetes (48% vs 50%) in HF infected with *Giardia* compared with non infected controls (Fig. 9b). Core microbiota analysis at the family level indicate different bacterial communities’ signatures between the two groups. In particular, the families *Lachnospiraceae* (20% vs 11%), *Ruminococcaceae* (12% vs 9%), *Lactobacillaceae* (7% vs 3%), were more abundant in *Giardia* infected HF mice while a decrease of *Bacteroidaceae* (13% vs 27%) was observed in those mice (Fig. 9a). The abundance of *Desulfovibrionaceae* and *Verrucomicrobiaceae*, represented by the genus *Akkermansia,* remained high in both group (13% and 14% respectively). Significant differences in biomarkers between HF and HF + G were confirmed by LDA score at the genus level (*p* < 0.05) (Fig. S4). Finally, comparison of LF and HF infected mice indicates a distinct separation between LF + G and HF + G groups (β-diversity metric; F-value: 5.0151; R-squared: 0.29475; *p* < 0.001) (Fig. S3). Shannon index (α-diversity) was significantly increased in HF infected mice versus LF + G mice (*p* < 0.05) (Fig. S3).

### HF diet enhances gut motility and increases stool water content in *Giardia* infected mice

**Figure 10:**
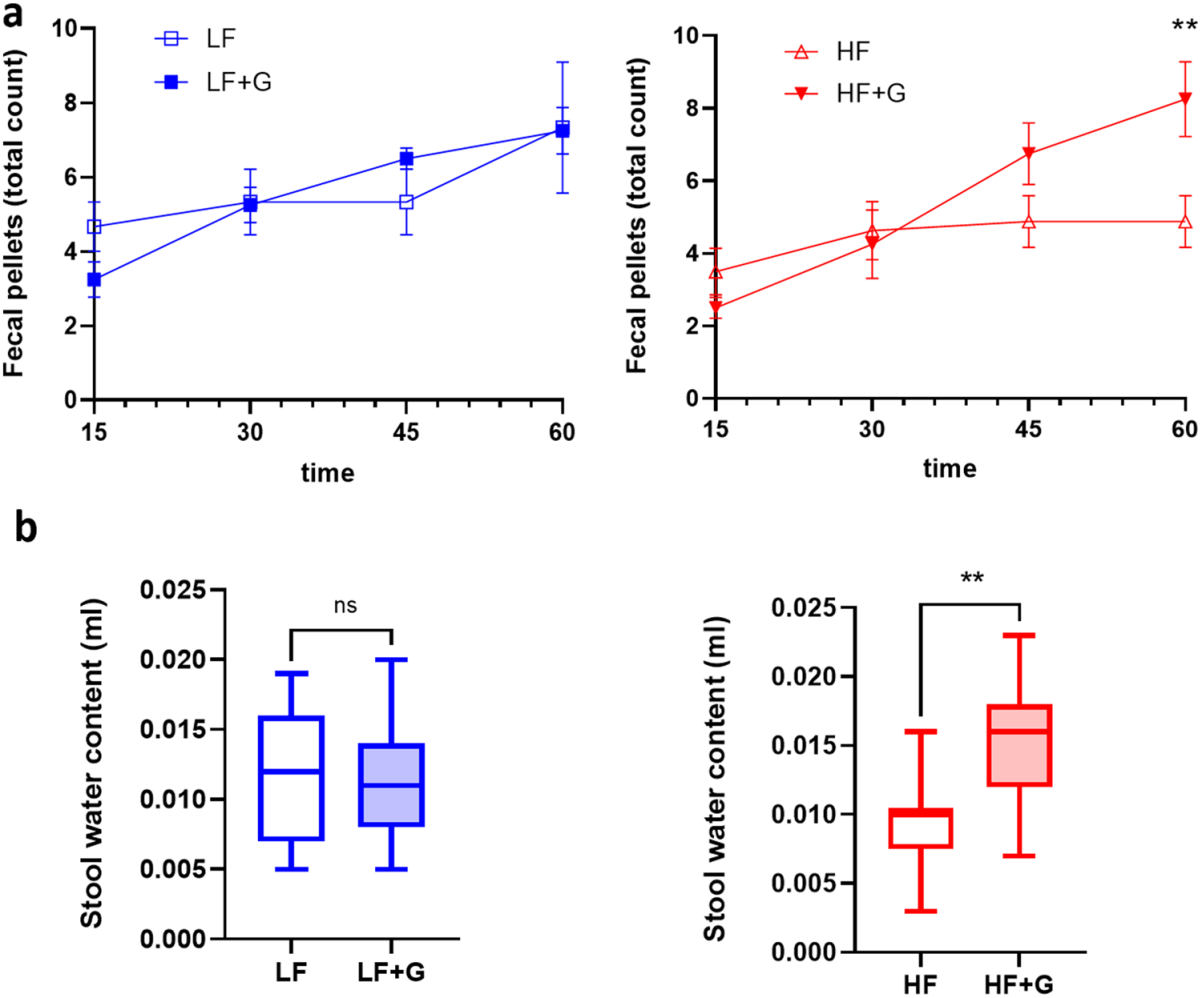
High fat exacerbates *Giardia*-induced increases in gut motility and stool water contents. (**a**) Colonic transit test measuring fecal pellet output. Total fecal pellet output per mouse over 1 h (time points: 15, 30, 45 and 60 min); n = 4 mice/group. (**b**) Water content of stools. Fresh fecal pellets were dried overnight at 60 °C and total stool water content was calculated by comparing wet/dry weight. n = 7–15 fecal pellets/group. LF = low fat control mice; LF + G = low fat infected mice; HF = high fat control mice; HF + G = high fat infected mice. Data are shown as box plots with min/max whiskers. ns = non-significant; **p < 0.01.

In view of the significant effects of dietary fat during a *Giardia* infection illustrated above, a final set of experiments assessed how these may correlate with consequences on gut motility and stool water content as markers of diarrheal disease. In mice given the HF diet, total fecal pellet count was increased at 45 min and 60 min when animals were infected with *Giardia* compared with non-infected animals (*p* < 0.01), indicative of increased intestinal motility (Fig. 10a). In animals given the LF diet, total fecal pellet counts were not different between control and infected animals. Moreover, *Giardia* infection did not alter water content in mice given the LF diet. In contrast, if animals were fed a HF diet, *Giardia* infection caused a significant increase in stool water content (*p* < 0.01) (Fig. 10b). The results indicate that a HF diet exacerbates *Giardia*-induced activation of gut motility and accumulation of water in the stools.

## Discussion

Nutrition and metabolically active tissues interact with immune cells and gut function to maintain homeostasis^20^. In giardiasis, nutrients and micronutrient deficiencies have been associated with immune disorders, malabsorption and higher infection rates via unknown mechanisms. In low-income countries, nutrient malabsorption in patients with giardiasis causes vitamin A deficiencies^47–50^. Conversely, *G. duodenalis* infection impairs vitamin A absorption in malnourished children^23^. Serum levels of other trace elements such as zinc and iron are decreased during giardiasis^28,31,32,51,52^. In this context, diet intervention and nutrient supplementation have been explored as a therapeutic strategy. Dietary supplementation with micronutrients appears to reduce parasitic loads and to attenuate diarrheal disease in children suffering from giardiasis^28,33^. Dietary fibers and prebiotic fibers have also been shown to play a role during *Giardia* infection. Upon *Giardia* infection, gerbils fed a high-fiber diet (insoluble fibers) exhibit lower trophozoite burden compared with infected gerbils fed a low-fiber diet^39^. Administration of the prebiotic fiber inulin to malnourished mice infected with *G. duodenalis* induces protective immune responses, restores weight gain, and diminishes *Giardia*-induced epithelial disruptions^42^. Additionally, protein deficiency decreases intestinal absorptive capacity by reducing villus length and increasing mucus production in response to *Giardia* infection^45^. Protein deficient diets increase parasite burden and exacerbate growth impairment in mice with giardiasis^53^, and a high protein diet promotes IL-23 production in bone marrow-derived cells^34^. Together, the data point to a crosstalk between nutrition and the detrimental effects of giardiasis, but the regulatory mechanisms remain obscure.

To date, few studies have investigated the role of dietary fats and other lipids in the outcome of *Giardia* infection. The present study investigated the effects of low fat (LF) and high fat (HF) diets on *Giardia* pathogenesis. The results demonstrate that a HF diet significantly increases parasite burden, exacerbates villus/crypt injury, and enhances mucosal infiltration of mixed inflammatory cells in giardiasis (the types of infiltrated inflammatory cells were not characterized). These changes were associated with elevated levels of pro-inflammatory cytokines and chemokines in infected mice given a high fat diet (*Il-6, Tnf-α*). Saturated (*i.e.,* palmitic acid) and monounsaturated (*i.e.,* oleic acid) fatty acids, which are more abundant in this HF diet formulation, significantly increased trophozoite metabolic activity and disrupted epithelial claudin-1 and claudin-4 in epithelial cells exposed to the parasite. The HF diet also exacerbated small intestinal goblet cell hyperplasia in association with elevated expression of the *Atoh1* gene, which regulates intestinal secretory cell proliferation, while in the colon it worsened the mucus granule depletion induced by the infection. The results also showed that consumption of either HF or LF diets induces significant shifts in the bacterial community of the gut. Microbiota dysbiosis induced by giardiasis was more pronounced in the mice given a HF diet, with significant increases in bacterial richness and evenness (α-diversity) and elevated representation of Firmicutes concurrent with reduced levels of Bacteroidetes. Moreover, in infected animals, the HF diet increased gut motility and stool water content, two factors implicated in the production of diarrhea. Together, these findings indicate that a HF diet, at least in part via increased saturated and monounsaturated fatty acids content (i.e., palmitic acid and/or oleic acid), significantly enhance the susceptibility to giardiasis. The observations are consistent with a recent report showing that short-term consumption of a HF diet increases host susceptibility to *Listeria monocytogenes* infection^54^.

Increased levels of IL-2, TNF-α, and IL-12 cytokines and LIX (CXCL5, RANTES) (Fig. 3a) chemokine in HF groups suggest that short-term HF diet induces low-grade inflammation in the small intestine by activating the NF-κB pathway and the recruiting M1 macrophages, consistent with previous observations^55–58^. The elevated *Tnf- α* and *Il-6* mRNA levels detected in HF + G mice are consistent with these changes. High concentrations of the chemotactic proteins MCP-1, MCP-2, and GM-CSF in HF groups are consistent with the increased infiltration of inflammatory cells observed in the histology of HF and HF + G mice (Figs. 2b, 3). Moreover, significantly increased CXCR2 gene expression in the HF + G group suggests neutrophils migrated to the site of infection; a phenomenon not observed in LF + G mice (Fig. 3b). Together, the data indicate that low-grade mucosal inflammation induced by a short-term consumption of HF diet is exacerbated upon *Giardia* infection. Interestingly, these results contrast with previous reports suggesting that *Giardia* can exert anti-inflammatory properties and immune evasion strategies via, at least in part, a cysteine-dependant cleavage of immune mediators^5,27,59^. Further research is warranted to determine weather diet-induced low-grade inflammation can supress these effects, and to assess the effect of fatty acid supplementation on *Giardia* cysteine protease activity.

Incubation of *Giardia* trophozoites with PA significantly increased their metabolic activity (Fig. 4). Similar results were obtained in the presence of OA (Fig. S8). These results suggest that the intake of dietary fat may directly promote the activity of *Giardia* trophozoites. Furthermore, PA worsened tight junctional (TJ) protein disruptions (ZO-1, claudin-1, and claudin-4) in *Giardia*-infected epithelial monolayers (Fig. 5). Disruption and rearrangement of intestinal epithelial cells TJ proteins by *Giardia* trophozoites have been well described^4, 7, 60^. Here, we suggest that TJ protein disruption following *Giardia* infection is enhanced in the presence of dietary PA and OA. Conversely, some dietary lipids such as gangliosides (e.g., milk fat) have been shown to exert anti-*Giardia* effects in vivo (in a *Giardia muris* rodent model) and in vitro (*G. duodenalis* isolate WB)^43^. Total milk fat in the diets used here was 6.05% in HF, and 1.05% in LF. The exact role of milk fat gangliosides in the observations reported here warrants further research. Analysis of lipids (*i.e*., triglycerides, free fatty acids and cholesterol) reported in other studies suggests that intestinal digestion of lipids is not impaired in suckling rats infected with *G. duodenalis* (isolate Paris 88/LCF/12)^40^. More research is needed to explore the growth-promoting effects of fatty acids on *Giardia* trophozoites, as well as the crosstalk between parasitism and lipid metabolism during infection in vivo. While lipid synthesis is limited in *Giardia*, fatty acids are key membrane and organelle constituents of *Giardia* trophozoites, and major source of energy^61^. Fluorescent lipid labelling experiments showed that PA is incorporated into *Giardia* trophozoites nuclear envelope and the plasma membrane^61^.

Disruptions of the mucus lining combined with goblet cell hyperplasia have been recently identified as pathogenic markers in giardiasis^9^. Findings from the present study reveal a key role for altered *Muc2* and *Atoh1* gene expression in this phenomenon. Indeed, increased goblet cell counts in infected animals was exacerbated by the HF diet (Fig. 6a,b), in association with elevated *Muc2* and *Atoh1* gene expression (Fig. 6c,d). These results are consistent with a previous report that indicated that these genes were implicated in the development of goblet cell hyperplasia and mucus hypersecretion^62^.

The gut microbiota has been shown to directly determine host’s susceptibility and/or resistance to colonization and persistence of *G. duodenalis* in mice^63–66^. *Giardia* causes dysbiosis, disrupts the microbiota biofilm overlaying the mucosa, and promotes the release of pathobionts from commensal communities, in turn leading to functional abnormalities in the gut^10,13^. In the present study, Bray–Curtis dissimilarity index (β-diversity metric) and Shannon and Simpson’s α-diversity showed a clear separation between HF and HF + G groups (Fig. 8). The altered relative abundance of bacterial species (at the phylum level) revealed an increase of Firmicutes and a decrease of Bacteroidetes, whereas no significant dysbiosis was observed between LF and LF + G mice (Fig. 9). In animals given the LF diet, the infection significantly increased lactobacilli abundance (LDA score > 5), an effect also observed in HF diet but to a lesser extent (Fig. S4d). Lactobacilli have recently been associated with a protective effect against *Giardia* infection^12,67,68^.

In uninfected animals, short-term consumption of LF or HF diet leads to a clear separation of bacteria communities as indicated by distinct β-diversity metrics, while species richness and evenness were not significantly altered (Fig. S9a, b). At the phylum level, HF diet was associated with an increase of Bacteroides and Verrucomicrobia and a decrease of Firmicutes relative to LF diet (Fig. S4, Fig. S9d). This diet-driven dysbiosis observed in our study is in accordance with previous reports showing that short-term consumption of LF/HF diet is sufficient to induce a shift of gut microbial communities^54,69^. Based on these observations, we hypothesize that short-term consumption of HF diet increases the host’s susceptibility to *Giardia* infection, and that the effects coincide with exacerbated microbiota dysbiosis, and a possible loss of the protective benefits of lactobacilli.

Finally, fecal pellet output was reduced in HF mice compared to LF mice, consistent with previous reports suggesting that HF diet delays colonic transit^70,71^. Upon infection, gut motility remained unchanged between LF and LF + G, whereas HF + G exhibited significantly increased gut motility (Fig. 10a). Moreover, stool water content was significantly increased in HF + G mice (Fig. 10b). The results suggest that *Giardia*-induced alterations in gastrointestinal motility and stool water contents are exacerbated by a HF diet.

In summary, findings from this study suggest that short-term consumption of a westernized high fat diet may increase the severity of giardiasis at least in part by promoting the growth of *Giardia* trophozoites through increased fatty acid exposure, and by predisposing to intestinal mucosal and epithelial damage. The effects of a HF diet during infection are associated with exacerbated gut microbiota dysbiosis and mucosal inflammation, as well as increased gut motility and stool water content. Altogether, this report puts in perspective the role of the diet in host-parasite interactions via a combination of gut pathophysiological phenomena, paving the way towards more research. Moreover, while this study assesses the influence of the diet at the peak of infection, future research will be needed to determine to the influence of the diet in post-giardiasis disorders such as IBS^2^. The findings also suggest that diet should be a consideration when developing new therapeutic approaches such as diet intervention.

## Materials and methods

### Animal studies

All experimental procedures were approved by the University Calgary Animal Care Committee under protocol AC17-0096 in compliance with the Canadian Council on Animal Care guidelines. This study has been designed and analyzed in accordance with ARRIVE guidelines. Male C57BL/6 mice, aged 3 to 4 weeks, were obtained from Charles River Laboratories (St-Constant, Quebec, Canada). Mice were fed for either 12 days (experimental condition #1) or 3 weeks (experimental condition #2) with either 5TJN/9GH3 Western high-fat (HF) diet (39.9% of the total energy from fat) or 5TJS/9GH4 low-fat (LF) diet (12.2% of the total energy from fat) prior to infection (TestDiet, USA) (Fig. S6). Inulin in the commercial formulation of the diets was replaced in both diets by cellulose to avoid any direct anti-*Giardia* effects of prebiotic fibers as described previously^42,72^. 3 to 4-week-old C57BL/6 mice were fed the LF or HF diets for 12 days prior to infection (experimental condition #1) to avoid common chronic obesity-related comorbidities and to allow robust infection^54,73,74^. Mice were orally infected with either *Giardia duodenalis* isolate GS/M trophozoites (1 × 10^7^ trophozoites in 100 µl of PBS; experimental condition #1) or *Giardia muris* cysts (1 × 10^4^ cysts in 100 µl of PBS; experimental condition #2). Control mice were orally administered 100 µl of sterile vehicle PBS. Mice were weighed daily and euthanized by cervical dislocation at day 7 post-infection. Small intestines were collected, and proximal jejunal Sects. (3 cm) were cut longitudinally and placed in tubes with 3 ml of ice-cold PBS for 20 min and vortexed. Live trophozoites were counted using a hemocytometer (mobility of flagella used as a marker of viability). Jejunal and distal colonic (1 cm) sections were collected and preserved by snap-freezing for RNA and protein analysis. Jejunal Sects. (1 cm) were collected in Formalin (10%) for histological analysis and fluorescent staining. Distal colonic Sects. (1 cm) were placed in Carnoy’s fixative for assessment of mucus granules (Alcian Blue/PAS staining) as previously described^24^.

### *G. duodenalis culture for *in vitro* and *in vivo* experiments*

*G. duodenalis* isolate NF (Assemblage A) was isolated from a giardiasis outbreak in Newfoundland, Canada^75^. *G. duodenalis* isolate GS/M (Assemblage B) was purchased from ATCC (Manassas, VA)*.* Trophozoites were cultured axenically in polystyrene conical tubes with TY1-S-33 medium supplemented with penicillin–streptomycin (Sigma-Aldrich) and used at peak culture density. For animal studies, *G. duodenalis* GS/M cultures were grown to confluence, placed on ice to detach trophozoites and washed with ice-cold PBS. Trophozoite cultures were prepared to a final concentration of 1 × 10^8^ trophozoites/ml in PBS. *G. muris* was kindly provided by Dr. Peter Geldhof (Ghent University, Ghent, Belgium). *G. muris* was passaged in female CD-1 mice (Charles River) until needed. Infected CD-1 mice were placed in empty cages and fasted for 3 h. Fecal pellets were further collected for cyst extraction by sucrose (1 M) gradient flotation and stored in cold PBS until ready to use.

### Multiplex cytokine analysis of mouse intestinal tissues

Snap-frozen mouse jejunal tissues were thawed on ice and homogenized in 1 ml of lysis buffer (T-PER) 20:1 buffer:tissue ratio (ThermoFischer Scientific). Protein concentration was measured using BCA protein kit (ThermoFischer Scientific) and normalized to 400 µl/ml. Cytokine expression levels were assessed via a Luminex Discovery Assay (Eve Technologies, Calgary, Alberta, Canada) according to the manufacturer’s protocol.

### RNA extraction and quantitative RT-PCR analysis

Small intestinal (jejunum) tissues were thawed and placed in a screw cap microfuge tube containing 600 µl of RLT buffer and 2-Mercaptoethanol (1 µl per ml) solution. Metal beads (0.5 mm) were added, and samples were shaken using FastPrep at a speed of 6.5 m/s, 2 × 60 s, with 5 min breaks on ice between each run. Supernatants were centrifuged at 14,000 g for 10 min at 4 °C, and mixed with 1 volume of 70% EtOH by pipetting. Total RNA was extracted using RNeasy Mini Kit (Qiagen) according to manufacturer protocols. RNA was quantified using a NanoDrop, and complementary DNA was synthesized using QuantiTect Reverse Transcription (RT) Kit (Qiagen). Quantitative polymerase chain reactions (qPCR) were performed with QuantiTect SYBR Green kit (Qiagen) on a Rotor Gene 3000 Cycler (Qiagen). Levels of mRNA expression of immune factors and mucin related genes were assessed using previously published primer sequences (Supplementary Table S1)^9,76–81^. Primers and reaction conditions are given in Supplementary Table S1 (primers were synthesized by University Core DNA Services at the University of Calgary, Canada). Data were analyzed by 2^-ΔΔCt^ method using β-actin as housekeeping gene and expressed as a fold change compared with control groups (LF and HF, respectively).

### Resazurin metabolic activity assays

The effect of palmitic acid (PA; C16:0) or oleic acid (OA; C18:10) on *Giardia* trophozoite metabolic activity was determined by resazurin assay in vitro as described previously with some modifications^82^. *G. duodenalis* strain NF was used instead of GS/M strain for this assay since GS/M isolate exhibited slower growth rate in 96-well plate setting. Briefly, *G. duodenalis* NF trophozoites were seeded in a 96-well microplate (10^5^ trophozoites per well) and incubated at 37 °C with PA. PA solutions were prepared as described above and diluted in TYI-S33 to reach working concentrations (0.05, 0.1, 0.25 mM). Control trophozoites received TYI-S33 + BSA (concentration of BSA was adjusted according to each dilution factor) for 24 h. After incubation, resazurin (10 μg/ml) was added to the wells and plates were further incubated at 37 °C for 90 min. Resorufin fluorescence was read using SpectraMax microplate reader (Molecular Devices Corporation, USA; λexc = 550 nm, λem = 590 nm).

### Fatty acid assays on SCBN cell lines

To further determine how a high fat diet may affect pathophysiology, effects of purified fatty acids on *Giardia*-epithelial cell interactions were assessed using a model of SCBN intestinal epithelial cells as previously described^46^. SCBN cells are canine duodenal nontumorigenic intestinal epithelial cell lines suitable for parasite-epithelial interactions (obtained from Dr. Pang laboratory, University of Newcastle, Australia)^83,84^. Palmitic acid (PA) or oleic acid (OA) were solubilized in 95% EtOH (0.1 M) and subsequently combined with a solution of Krebs–Ringer Bicarbonate buffer (KRB) and low-endotoxin BSA (5%) to a reach a concentration of 2.5 mM. To allow BSA to conjugate with PA, the solution was incubated at 37 °C for 24 h. Following incubation, the solution was diluted in DMEM to reach a working concentration of PA of 0.3 mM. In parallel, a control solution (Ctrl) of DMEM supplemented with KRB and low-endotoxin BSA but devoid of PA was prepared for assays. Fatty acid–BSA complex solutions were freshly prepared before each experiment. SCBN cells were seeded at 1 × 10^4^ cells per ml in chamber slides (ThermoFischer Scientific) and incubated at 37 °C, 5% CO_2_. At confluence, cells were incubated for 2 h at 37 °C, 5% CO_2_ with either the Ctrl, the PA or OA solution. Subsequently, cells were exposed to *G. duodenalis* GS/M (MOI = 10:1) or left un-infected and incubated at 37 °C, 5% CO_2_ for 24 h. For staining, *Giardia* trophozoites were first detached from SCBNs using 200μL of DMEM supplemented with 0.2% formononetin (Sigma-Aldrich), as previously described^75,85^. Cells were washed 3 times with ice-cold PBS to ensure complete removal of trophozoites. This step was also performed on non-infected cells. Cells were fixed using an ice-cold methanol:acetone (1:1) solution at -20 °C for 20 min, followed by washing with PBS for 30 min at room temperature (RT), replacing wash every 10 min. To minimise non-specific binding of primary and secondary antibodies in samples, a PBS blocking solution containing 1% BSA was incubated on cells for 30 min at RT. Following blocking, primary antibodies were incubated on cells overnight at 4 °C (1:1000 dilution) and washed 3 times with ice-cold PBS. Appropriate secondary antibodies were then added for 1 h at RT and washed 3 times as per the primary antibody step. Finally, slides were sealed and observed using a Leica DMR fluorescent microscope (Leica, Wetzlar, Germany). Images were taken using a Retiga 2000x (Q imaging, Surrey, BC, Canada) camera and analyzed on ImageJ. For immunohistochemistry, the following antibodies were used: ZO-1 (Abcam; polyclonal, rabbit), claudin-1 (Invitrogen; polyclonal, rabbit), claudin-4 (Invitrogen; polyclonal, rabbit), AlexaFluor 488 (goat anti-rabbit) and AlexaFluor 594 (goat anti-rabbit).

### Histological analysis and PAS staining

Formalin-fixed paraffin-embedded jejunal intestine tissues were cut into 4 μm sections and stained using hematoxylin and eosin to assess cellular and tissue structure in the small intestine. Crypt-villus ratios were determined (blinded) by measuring the crypt and villus height on ImageJ software (National Institutes of Health) as described previously with minor modifications^86^. Results were expressed as the average of 7 to 10 crypt-villus units.

Histological score was determined (blinded) on jejunal sections by assessing several disease parameters (cell infiltration, cell hyperplasia, goblet cell loss, cryptitis, epithelial erosion, ulceration, tissue granulation, irregular crypts, crypt loss, villous damage, and blunting). Carnoy’s-fixed and formalin-fixed paraffin-embedded small intestine and colonic tissues were cut into 4 µm sections and deparaffinized in xylene (3 times) and rehydrated several times in graded ethanol (100% and 95%). Tissues were then stained for mucins using Alcian Blue/PAS staining (Newcomer Supply) using the manufacturer’s protocol. Mucin grains and goblet cells (PAS +) were enumerated and reported as the average of goblet cells (PAS +) per 10 crypts as previously (blinded)^87^.

### Fecal microbiota analysis and Illumina 16S rRNA gene sequencing

Mouse fecal pellets were collected at day 7 Post Infection and stored at -80 °C until DNA extraction. Whole bacterial genomic DNA extraction procedure was adapted from Lamas et *al*.^88^. In brief, frozen fecal pellets were placed in a screw cap microfuge tube containing 250 µl of guanidine thiocyanate (4 M) Tris–HCl (pH7.5, 0.1 M) solution and 40 µl of N-Laurosyl Sarcosine 10% (Sigma-Aldrich). After thawing, 500 µl of N-Laurosyl Sarcosine 5% in Phosphate buffer (pH8, 0.1 M) were added and samples were incubated at 70 °C for 2 h. Silica glass beads (0.1 mm, 500 mg) were added, and samples were shaken using FastPrep at speed 6.5 m/s, 3 × 30 s, with 5 min breaks on ice between each run. PolyVinylPolyPyrrolidone (Sigma-Aldrich) (15 mg) was subsequently added to the samples and tubes were centrifuged for 5 min at max speed (20,000 g). Supernatants were collected, and lysed pellets were washed twice with TENP buffer (Tris–Cl, EDTA 0.5 M, NaCl 5 M, PVPP 1%). Supernatants were combined and an extract centrifugation step was performed to remove debris. Ice-cold isopropanol was added to the supernatant, and samples were mixed gently and incubated 10 min at room temperature and centrifuged for at 20,000 g for 10 min at room temperature. Pellets were resuspended in 450 µl of Phosphate buffer (pH8 0.1 M) and 50 µl of potassium acetate (5 M), and incubated at 60 °C for 10 min. Samples were placed at 4 °C over night and centrifuged at 20,000 g for 30 min. DNA was then purified by adding RNAse (2 µl, 10 mg/ml) for 30 min at 37 °C. DNA were precipitated by adding ice cold absolute ethanol (1 ml) and sodium acetate (50 µl, 3 M) and centrifuging 10 min at 20,000 g. Pellets were washed several times by adding 70% Ethanol (1 ml) to the pellets. DNA pellets were then dried at room temperature and resuspended in 100 µl of TE buffer (1X), and stored O.N. at 4 °C and -20 °C for long term storage. DNA was further purified using DNA clean and concentrator kit (Zymo Research).

Amplicon sequencing libraries were obtained from the V3-V4 region of the 16S SSU rRNA gene using 515F-806R primers. Paired-end amplicons (250 bases) were sequenced on Illumina Mi-Seq (Genome Québec Innovation Centre, Montreal, Canada). Bioinformatics analyses were performed using Microbiome analyst online software. The 16S rRNA amplicons were clustered into operational taxonomic units (OTU) with a 97% identity threshold. Data are represented using Total Sum Scaling (proportional abundance of species) to remove sequencing-related technical biases. One sample from HF *Giardia* group was removed due to low abundance OTUs and failure to pass the OTU selection step. The β-diversity among microbial communities was assessed using Bray–Curtis dissimilarity index and visualized through Principal Coordinate Analysis (PCoA) plot. The α-diversity was assessed by calculating both Shannon and Simpson diversity indexes. Core microbiota representation was used to characterized bacterial taxa at the family level based on their prevalence at a given abundance threshold (relative abundance).

### Gut motility assays and assessment of stool water content

As *Giardia*-induced diarrhea is associated with increased intestinal motility^89^, additional experiments measured the effects of the diets on gut motility and stool water content. Before sacrifice, mice from each group (LF, LF + G, HF, HF + G) were placed in individual cages without water and food for 60 min and fecal pellet number was counted every 15 min for each mouse (n = 4 per group). Fecal pellets were collected at 60 min and placed in an empty Eppendorf tube. Fecal pellets were dried overnight at 60 °C and water content of stools was determined by comparing wet/dry weight difference as an indicator of loose or watery stools.

### Statistical analysis of data

Statistical analysis was performed using GraphPad Prism software (GraphPad Prism 8, La Jolla, USA). The normality of the data was assessed before statistical analysis. Comparison between groups with normal distribution was performed using one-way analysis of variance (ANOVA) followed by Tukey’s test for multiple comparison analyses. Groups with non-parametric data were analyzed using Kruskal–Wallis test. Comparison of two sets of data with a Gaussian distribution was done using Student's t-test (unpaired), and Mann–Whitney’s test was used to compare two sets of non-parametric data. Identification of outliers was performed using Grubbs' test (α = 0.05). Error bars represent standard error of the mean (histograms) or min–max (box-plot). All centre values are median for boxplots and mean for histograms. *P* values of less than 0.05 were considered statistically significant.

### Statement of ethics

All experiments involving rodents have been approved by the Animal Care Committee at the University of Calgary (approval certificate #AC17-0096). The committee approved of the procedures described in the protocol and certified that they are in accordance with the principles outlined in the current guidelines of the Canadian Council on Animal Care.

## Supplementary information

Supplementary Information.
