# Supplementary material for: High-fat diet increases the severity of Giardia infection in association with low-grade inflammation and gut microbiota dysbiosis
Source: Sci Rep. 2021 Sep 22;11:18842. doi: 10.1038/s41598-021-98262-8 (PMC8458452; doi:10.1038/s41598-021-98262-8)
Supplement: Supplementary file 1 — Supplementary Information. [file 41598_2021_98262_MOESM1_ESM.pdf]

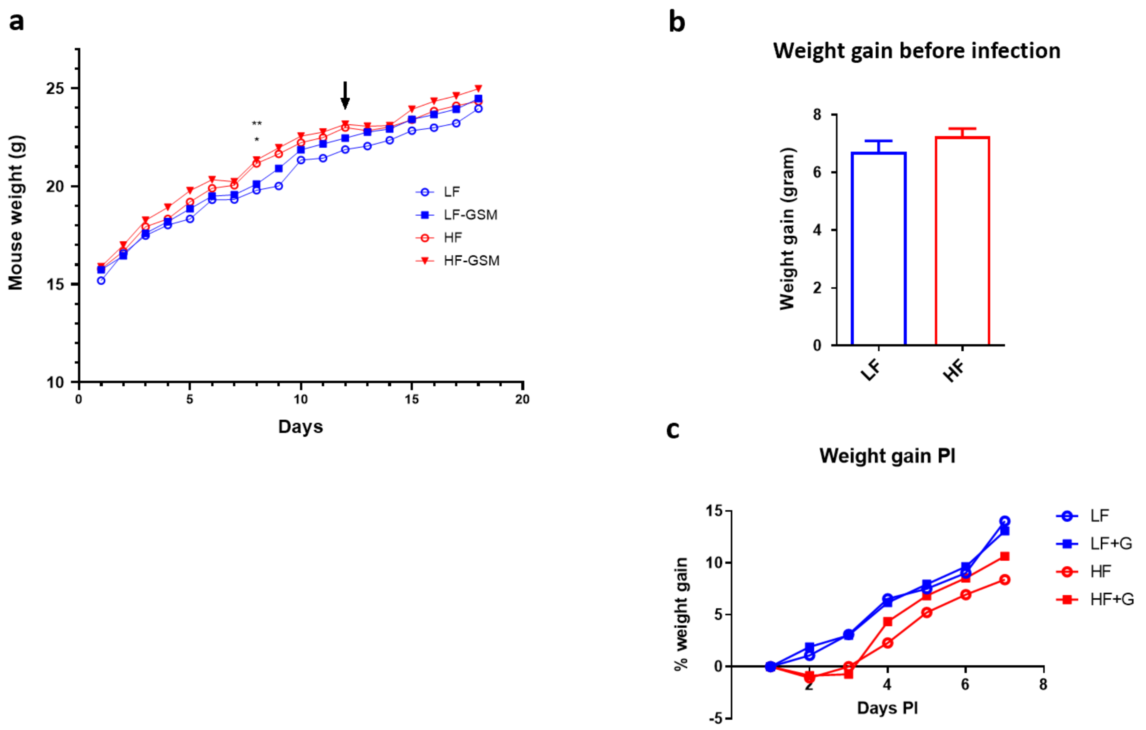

Figure S1. Short term consumption of HF diet does not significantly increase body weight gain. (a) total mouse weight (experimental condition #1) (day 1 to day 18). Mouse were infected with *G. duodenalis* GS/M at day 12 (represented by a black arrow). (b) Weight gain in grams before infection. (c) weight gain at day 7 post infection. n=8-9/group. LF=low fat control mice; LF+G= low fat infected mice; HF= high fat control mice; HF+G= high fat infected mice. *$p < 0.01$; **$p < 0.01$.

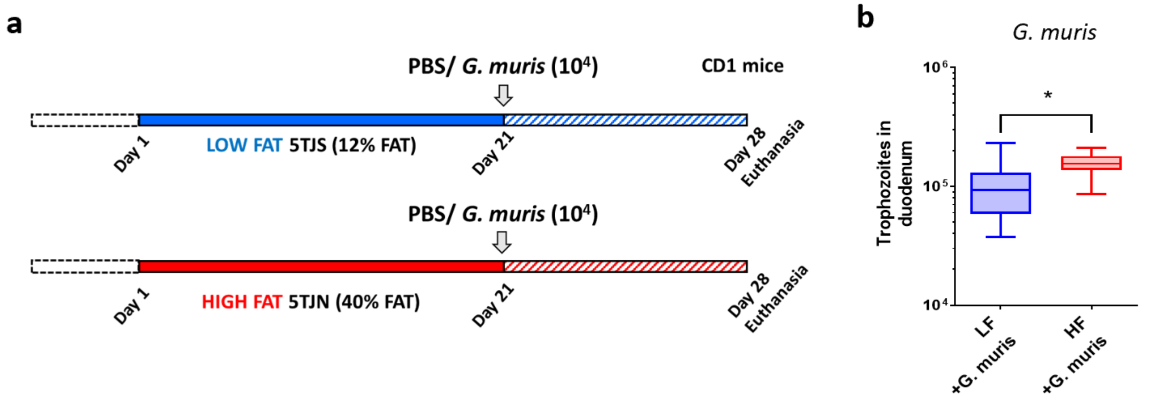

c

Days after infection with *Giardia*	*G. muris*	
	Low Fat	High Fat
Number of infected animals 7 days P.I.	8/8	7/7
Trophozoite burden (duodenum)	$1.02 \times 10^5 \pm 0.21$	$1.53 \times 10^5 \pm 0.14$

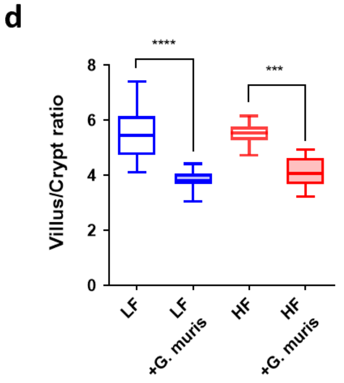

Figure S2. High dietary fat increases host susceptibility to *G. muris* infection in CD1 mice. (a) Experimental design (experimental condition #2)**.** CD-1 mice (4-5 week-old) received either a low fat (LF) or a high fat (HF) diet for 21 days and were infected with *Giardia muris* (10^4 cysts). Control animals received PBS. Animals were euthanized at 7 days post-infection (day 28). (b) Duodenal *G. duodenalis* trophozoite burden was assessed at 7 days PI. Trophozoite burden was higher in HF+G. muris mice compared with LF+G. muris mice. No trophozoites were detected in the PBS group. (c) Number of infected animals per experimental group at 7 days PI. (d) Morphometric measurements of the jejunal mucosa. The figure illustrates villus height to crypt depth ratios at day 7 post infection. LF=low fat control mice; LF+G. muris= low fat infected mice; HF= high fat control mice; HF+G. muris= high fat infected mice. Data are representative of 8 mice/group and are shown as box plots with min/max whiskers; ****p< 0.001.

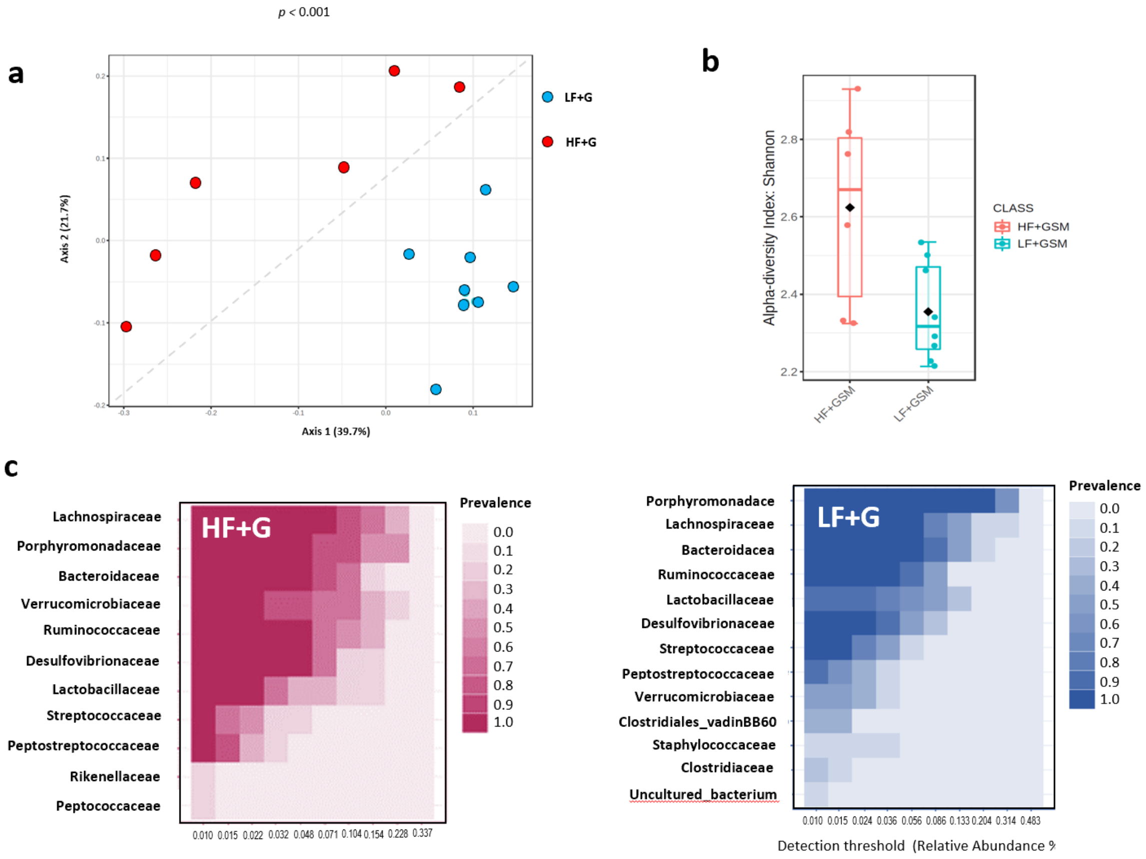

Figure S3. α-, β- diversity and core microbiota comparison between LF+G and HF+G mice. The fecal microbiota composition of *Giardia*-infected LF+G group was investigated at day 7 PI and compared with HF+G group. Taxonomic identification of the gut microbiota was assessed via 16S rRNA gene sequencing using Illumina MiSeq platform. The 16S rRNA amplicons were clustered into operational taxonomic units (OTU) with a 97% identity threshold. Data are represented using Total Sum Scaling (proportional abundance of species) to remove sequencing-related technical biases. (a) The β-diversity between LF+G (blue dots) and HF+G (red dots) microbial communities was assessed using Bray-Curtis dissimilarity index and visualized through Principal Coordinate Analysis (PCoA) plot. (b) The α-diversity was assessed by calculating Shannon diversity index. Data are expressed as box plots with min/max whiskers;*$p < 0.05$. (c)

Bacterial taxa at the family level based on their prevalence at a given abundance threshold (core microbiota representation; relative abundance %); prevalence scale ranges from 0.0 (white) to 1.0 (dark fuchsia or blue). n=6-8 mice per group; LF+G=low fat infected mice; HF+G= high fat infected mice.

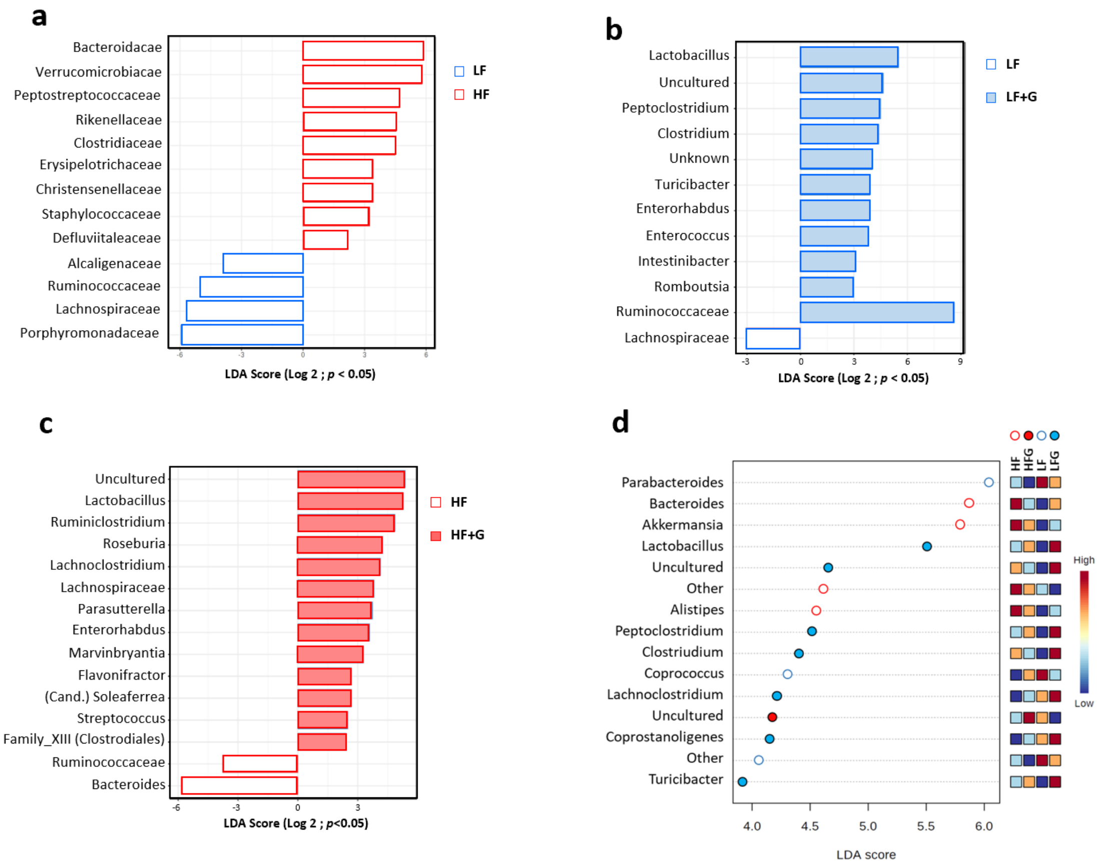

Fig S4. Linear Discriminant Analysis scores for each experimental condition. Linear Discriminant Analysis (LDA) scores of the different taxa (family and genus levels) were calculated for each experimental condition. Negative and positive LDA scores represent bacterial groups overrepresented in the corresponding group. (a) LDA scores between LF and HF; (b) LDA scores between LF and LF+G groups; (c) LDA scores between HF and HF+G groups; LDA scores are expressed as Log 2 scale; *p*<0.05. (d) LDA score showing the most overrepresented bacterial taxa among LF, LF+G, HF, HF+G groups (scale= 4 to 6).

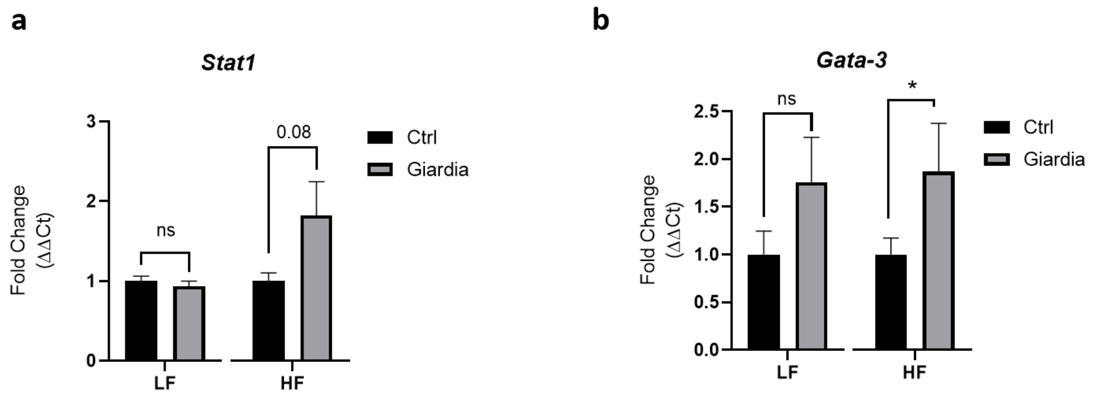

Fig S5. Jejunal *Stat1* and *Gata-3* gene expression in all experimental conditions.

Jejunal mRNA levels of (a) *Stat1* and (b) *Gata-3* were measured by Real-Time Quantitative Reverse Transcription-PCR) and normalized to β-actin mRNA ($2^{-\Delta\Delta Ct}$ method). Data are represented as fold change compared to LF and HF uninfected controls, respectively; n=8-9 per group. LF=low fat control mice; LF+G= low fat infected mice; HF= high fat mice; HF+G= high fat infected mice. Data are shown as mean ± S.E.M; *$p < 0.05$. ns= non-significant

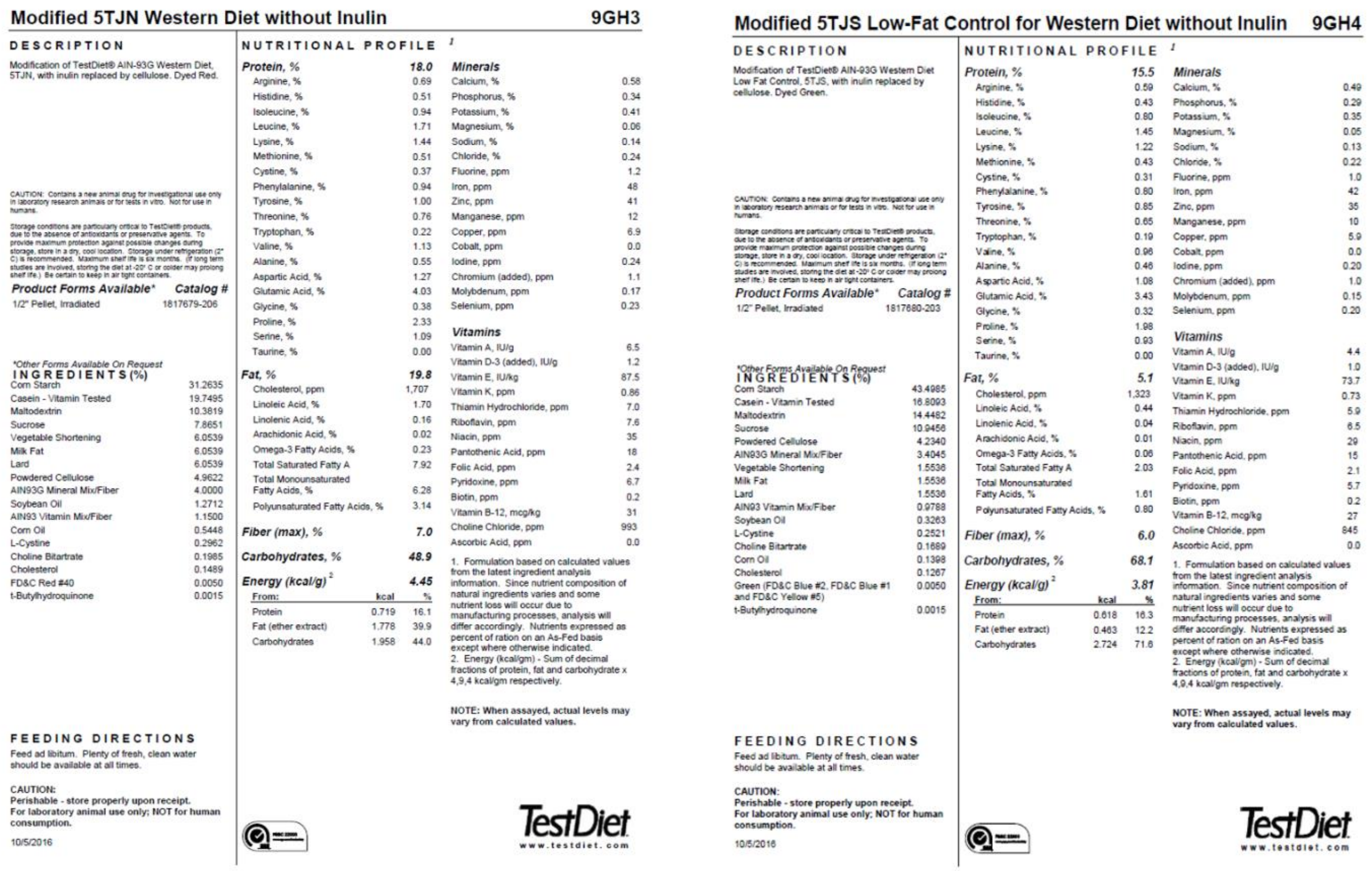

Modified 5TJN Western Diet without Inulin — 9GH3

DESCRIPTION

Modification of TestDiet® AIN-93G Western Diet, 5TJN, with inulin replaced by cellulose. Dyed Red.

CAUTION: Contains a new animal drug for investigational use only in laboratory research animals or for tests in vitro. Not for use in humans.

Storage conditions are particularly critical to TestDiet® products, due to the absence of antioxidants or preservative agents. To provide maximum protection against possible changes during storage, store in a dry, cool location. Storage under refrigeration (2° C) is recommended. Maximum shelf life is six months. (If long term studies are involved, storing the diet at -20° C or colder may prolong shelf life.) Be certain to keep in air tight containers.

*Product Forms Available**	*Catalog #*
1/2" Pellet, Irradiated	1817679-206

*Other Forms Available On Request

INGREDIENTS (%)

Corn Starch	31.2635
Casein - Vitamin Tested	19.7495
Maltodextrin	10.3819
Sucrose	7.8651
Vegetable Shortening	6.0539
Milk Fat	6.0539
Lard	6.0539
Powdered Cellulose	4.9622
AIN93G Mineral Mix/Fiber	4.0000
Soybean Oil	1.2712
AIN93 Vitamin Mix/Fiber	1.1500
Corn Oil	0.5448
L-Cystine	0.2962
Choline Bitartrate	0.1985
Cholesterol	0.1489
FD&C Red #40	0.0050
t-Butylhydroquinone	0.0015

NUTRITIONAL PROFILE [1]

Protein, %	***18.0***
Arginine, %	0.69
Histidine, %	0.51
Isoleucine, %	0.94
Leucine, %	1.71
Lysine, %	1.44
Methionine, %	0.51
Cystine, %	0.37
Phenylalanine, %	0.94
Tyrosine, %	1.00
Threonine, %	0.76
Tryptophan, %	0.22
Valine, %	1.13
Alanine, %	0.55
Aspartic Acid, %	1.27
Glutamic Acid, %	4.03
Glycine, %	0.38
Proline, %	2.33
Serine, %	1.09
Taurine, %	0.00
Fat, %	***19.8***
Cholesterol, ppm	1,707
Linoleic Acid, %	1.70
Linolenic Acid, %	0.16
Arachidonic Acid, %	0.02
Omega-3 Fatty Acids, %	0.23
Total Saturated Fatty A	7.92
Total Monounsaturated Fatty Acids, %	6.28
Polyunsaturated Fatty Acids, %	3.14
Fiber (max), %	***7.0***
Carbohydrates, %	***48.9***
Energy (kcal/g) [2]	***4.45***

From:	kcal	%
Protein	0.719	16.1
Fat (ether extract)	1.778	39.9
Carbohydrates	1.958	44.0

Minerals	
Calcium, %	0.58
Phosphorus, %	0.34
Potassium, %	0.41
Magnesium, %	0.06
Sodium, %	0.14
Chloride, %	0.24
Fluorine, ppm	1.2
Iron, ppm	48
Zinc, ppm	41
Manganese, ppm	12
Copper, ppm	6.9
Cobalt, ppm	0.0
Iodine, ppm	0.24
Chromium (added), ppm	1.1
Molybdenum, ppm	0.17
Selenium, ppm	0.23
Vitamins	
Vitamin A, IU/g	6.5
Vitamin D-3 (added), IU/g	1.2
Vitamin E, IU/kg	87.5
Vitamin K, ppm	0.86
Thiamin Hydrochloride, ppm	7.0
Riboflavin, ppm	7.6
Niacin, ppm	35
Pantothenic Acid, ppm	18
Folic Acid, ppm	2.4
Pyridoxine, ppm	6.7
Biotin, ppm	0.2
Vitamin B-12, mcg/kg	31
Choline Chloride, ppm	993
Ascorbic Acid, ppm	0.0

1. Formulation based on calculated values from the latest ingredient analysis information. Since nutrient composition of natural ingredients varies and some nutrient loss will occur due to manufacturing processes, analysis will differ accordingly. Nutrients expressed as percent of ration on an As-Fed basis except where otherwise indicated.
2. Energy (kcal/gm) - Sum of decimal fractions of protein, fat and carbohydrate x 4,9,4 kcal/gm respectively.

NOTE: When assayed, actual levels may vary from calculated values.

FEEDING DIRECTIONS

Feed ad libitum. Plenty of fresh, clean water should be available at all times.

CAUTION:
Perishable - store properly upon receipt.
For laboratory animal use only; NOT for human consumption.

10/5/2016

TestDiet
www.testdiet.com

Modified 5TJS Low-Fat Control for Western Diet without Inulin — 9GH4

DESCRIPTION

Modification of TestDiet® AIN-93G Western Diet Low Fat Control, 5TJS, with inulin replaced by cellulose. Dyed Green.

CAUTION: Contains a new animal drug for investigational use only in laboratory research animals or for tests in vitro. Not for use in humans.

Storage conditions are particularly critical to TestDiet® products, due to the absence of antioxidants or preservative agents. To provide maximum protection against possible changes during storage, store in a dry, cool location. Storage under refrigeration (2° C) is recommended. Maximum shelf life is six months. (If long term studies are involved, storing the diet at -20° C or colder may prolong shelf life.) Be certain to keep in air tight containers.

*Product Forms Available**	*Catalog #*
1/2" Pellet, Irradiated	1817680-203

*Other Forms Available On Request

INGREDIENTS (%)

Corn Starch	43.4985
Casein - Vitamin Tested	16.8093
Maltodextrin	14.4482
Sucrose	10.9456
Powdered Cellulose	4.2340
AIN93G Mineral Mix/Fiber	3.4045
Vegetable Shortening	1.5536
Milk Fat	1.5536
Lard	1.5536
AIN93 Vitamin Mix/Fiber	0.9788
Soybean Oil	0.3263
L-Cystine	0.2521
Choline Bitartrate	0.1689
Corn Oil	0.1398
Cholesterol	0.1267
Green (FD&C Blue #2, FD&C Blue #1 and FD&C Yellow #5)	0.0050
t-Butylhydroquinone	0.0015

NUTRITIONAL PROFILE [1]

Protein, %	***15.5***
Arginine, %	0.59
Histidine, %	0.43
Isoleucine, %	0.80
Leucine, %	1.45
Lysine, %	1.22
Methionine, %	0.43
Cystine, %	0.31
Phenylalanine, %	0.80
Tyrosine, %	0.85
Threonine, %	0.65
Tryptophan, %	0.19
Valine, %	0.96
Alanine, %	0.46
Aspartic Acid, %	1.08
Glutamic Acid, %	3.43
Glycine, %	0.32
Proline, %	1.98
Serine, %	0.93
Taurine, %	0.00
Fat, %	***5.1***
Cholesterol, ppm	1,323
Linoleic Acid, %	0.44
Linolenic Acid, %	0.04
Arachidonic Acid, %	0.01
Omega-3 Fatty Acids, %	0.06
Total Saturated Fatty A	2.03
Total Monounsaturated Fatty Acids, %	1.61
Polyunsaturated Fatty Acids, %	0.80
Fiber (max), %	***6.0***
Carbohydrates, %	***68.1***
Energy (kcal/g) [2]	***3.81***

From:	kcal	%
Protein	0.618	16.3
Fat (ether extract)	0.463	12.2
Carbohydrates	2.724	71.6

Minerals	
Calcium, %	0.49
Phosphorus, %	0.29
Potassium, %	0.35
Magnesium, %	0.05
Sodium, %	0.13
Chloride, %	0.22
Fluorine, ppm	1.0
Iron, ppm	42
Zinc, ppm	35
Manganese, ppm	10
Copper, ppm	5.9
Cobalt, ppm	0.0
Iodine, ppm	0.20
Chromium (added), ppm	1.0
Molybdenum, ppm	0.15
Selenium, ppm	0.20
Vitamins	
Vitamin A, IU/g	4.4
Vitamin D-3 (added), IU/g	1.0
Vitamin E, IU/kg	73.7
Vitamin K, ppm	0.73
Thiamin Hydrochloride, ppm	5.9
Riboflavin, ppm	6.5
Niacin, ppm	29
Pantothenic Acid, ppm	15
Folic Acid, ppm	2.1
Pyridoxine, ppm	5.7
Biotin, ppm	0.2
Vitamin B-12, mcg/kg	27
Choline Chloride, ppm	845
Ascorbic Acid, ppm	0.0

1. Formulation based on calculated values from the latest ingredient analysis information. Since nutrient composition of natural ingredients varies and some nutrient loss will occur due to manufacturing processes, analysis will differ accordingly. Nutrients expressed as percent of ration on an As-Fed basis except where otherwise indicated.
2. Energy (kcal/gm) - Sum of decimal fractions of protein, fat and carbohydrate x 4,9,4 kcal/gm respectively.

NOTE: When assayed, actual levels may vary from calculated values.

FEEDING DIRECTIONS

Feed ad libitum. Plenty of fresh, clean water should be available at all times.

CAUTION:
Perishable - store properly upon receipt.
For laboratory animal use only; NOT for human consumption.

10/5/2016

TestDiet
www.testdiet.com

Figure S6. Diet formulation. Composition of 5TJN/9GH3 Western high-fat (HF) diet and 5TJS/9GH4 low-fat (LF) diet (TestDiet, USA).

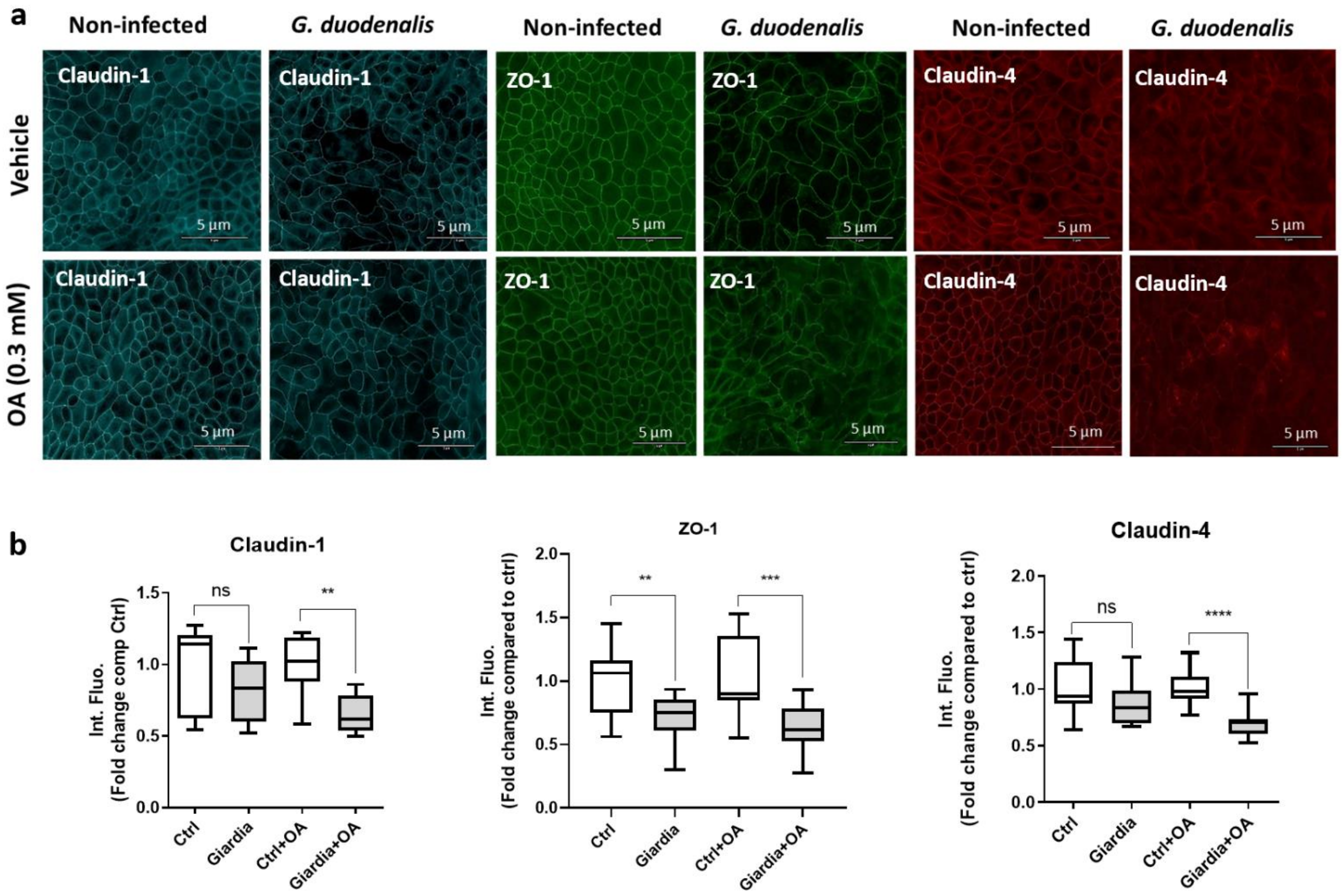

Figure S7. Disruption of tight junction proteins claudin-1 and claudin-4 by *Giardia* is enhanced in presence of oleic acid in SCBN cells. Intestinal epithelial cells **(**SCBN) were pre-treated with vehicle (Ctrl) or oleic acid (OA; 0.3 mM) and challenged for 24 hours with *G. duodenalis* isolate GS/M (*Giardia*+OA; MOI=10:1) or left uninfected (Ctrl+OA). (a) SCBN cells were probed for immunofluorescence staining of ZO-1, claudin-1 and claudin-4 proteins (scale bar = 5 µm). (b) ZO-1, claudin-1 and claudin-4 relative protein levels were quantified by fluorescence intensity compared with DAPI (ImageJ) and expressed as fold change compared to Ctrl group. n= 4 per group. Data are expressed as box plots with min/max whiskers. *$p < 0.05$, **$p < 0.01$, ***$p < 0.001$, ****$p < 0.0001$.

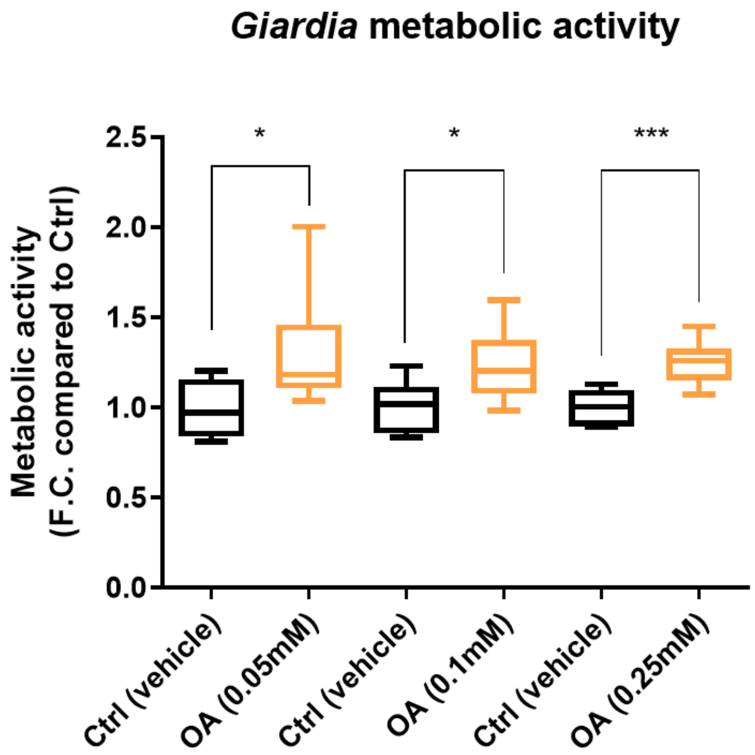

Figure S8. Oleic acid enhances *Giardia* trophozoites metabolic activity. Levels of resorufin converted from resazurin by *G. duodenalis* trophozoites (isolate NF) at 37 °C for 90 min. Resorufin fluorescence was read at λexc = 550 nm; λem = 590 nm. Data are represented as a fold-change compared to control. Ctrl= control (vehicle); OA=Palmitic acid. Data are shown as box plots with min/max whiskers; *p< 0.05, ***p< 0.001

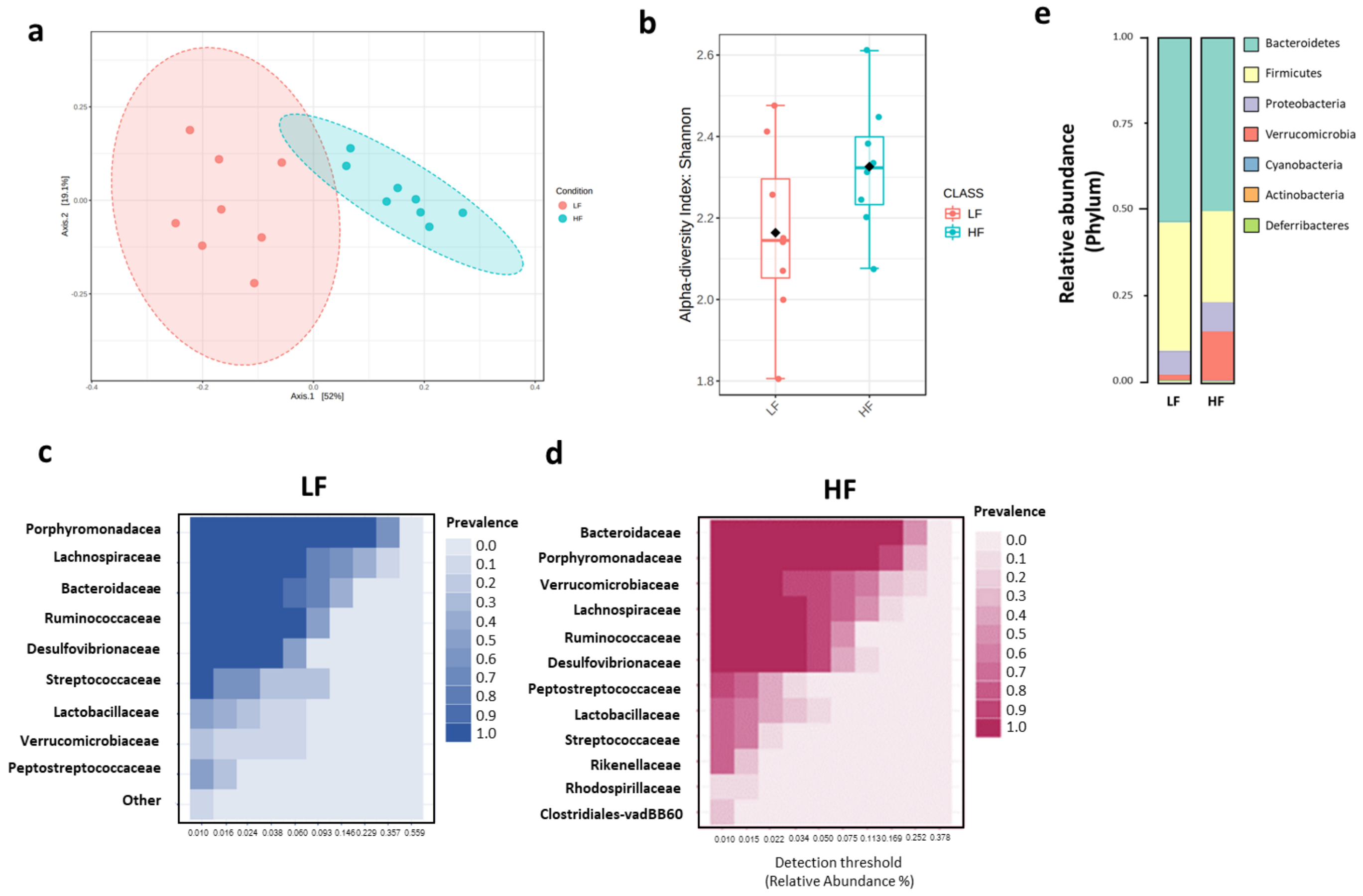

Figure S9. Microbiota composition of LF and HF uninfected mice. The fecal microbiota composition of uninfected HF group was investigated at day 7 post PBS administration and compared with LF control mice microbiota, respectively. Taxonomic identification of the gut microbiota was assessed via 16S rRNA gene sequencing using Illumina MiSeq platform. The 16S rRNA amplicons were clustered into operational taxonomic units (OTU) with a 97% identity threshold. Data are represented using Total Sum Scaling (proportional abundance of species) to remove sequencing-related technical biases. (a) The β-diversity between LF and HF microbial communities was assessed using Bray-Curtis dissimilarity index and visualized through Principal Coordinate Analysis (PCoA) plot. (b) The α-diversity was assessed by calculating Shannon

diversity index; data are expressed as box plots with min/max whiskers. n=8 mice per group; (c, d) Bacterial taxa at the family level based on their prevalence at a given abundance threshold (core microbiota representation; relative abundance %); prevalence scale ranges from 0.0 (white) to 1.0 (dark blue). (d) Bar charts representative of the relative abundance of microbial taxa at the phylum level between LF and HF groups (uninfected) ; LF=low fat control mice; HF= high fat control mice.

Gene of Interest	Forward Sequence	Reverse Sequence	Reference
Murine Muc2	5′-GAAGCCAGATCCCGAAACCA-3′	5′-CCAGCTTGTGGGTGAGGTAG-3′	9
Murine IL-10	5′- ATTTGAATTCCCTGGGTGAGAAG -3′	5′- CACAGGGGAGAAATCGATGACA -3′	72
Murine IL-6	5′- CCA GAA ACC GCT ATG AAG TTC C -3′	5′- TCA CCA GCA TCA GTC CCA AG -3′	71
Murine TNF-alpha	5′- CTC CAG GCG GTG CCT ATG T -3′	5′- GAA GAG CGT GGT GGC CC -3′	71
Murine Gata-3	5′- TTTACCCTCCGGCTTCATCCTCCT - 3′	5′- TGCACCTGATACTTGAGGCACTCT - 3′	69
Murine Atoh1	5′-AGCTTCCTCTGGGGGTTACT -3′	5′-TTCTGTGCCATCATCGCTGT-3′	68
Murine Stat1	5′- TGGGAAGTATTATTCCAGACCAAA-3′	5′-AGTCTTGATGTATCCAGTTCG-3′	70
Murine Cxcr2	5′- CAGCTGCCTTAACCCCATCA – 3′	5′- CTTGAGAAGTCCATGGCGAAA-3′	Designed in this study
β-actin	5′-GAAGTCCCTCACCCTCCCAA-3′	5′-GGCATGGACGCGACCA-3′	67

Table. S1. Sequences of primers (5’ -3’) used in this study.
